# Neuron Specific Rab4 Effector GRASP-1 Coordinates Membrane Specialization and Maturation of Recycling Endosomes

**DOI:** 10.1371/journal.pbio.1000283

**Published:** 2010-01-19

**Authors:** Casper C. Hoogenraad, Ioana Popa, Kensuke Futai, Emma Sanchez-Martinez, Phebe S. Wulf, Thijs van Vlijmen, Bjorn R. Dortland, Viola Oorschot, Roland Govers, Maria Monti, Albert J. R. Heck, Morgan Sheng, Judith Klumperman, Holger Rehmann, Dick Jaarsma, Lukas C. Kapitein, Peter van der Sluijs

**Affiliations:** 1 Department of Neuroscience, Erasmus Medical Center, Rotterdam, The Netherlands; 2 Department of Cell Biology, University Medical Center (UMC) Utrecht, Utrecht, The Netherlands; 3 The Picower Institute for Learning and Memory, Massachusetts Institute of Technology, Cambridge, Massachusetts, United States of America; 4 Cell Microscopy Center, UMC Utrecht, Utrecht, The Netherlands; 5 Department of Functional Genomics, Centre for Neurogenomics and Cognitive Research, VU Amsterdam, The Netherlands; 6 Department of Biomolecular Mass Spectrometry and Proteomics Group, Bijvoet Centre for Biomolecular Research and Utrecht Institute for Pharmaceutical Sciences, Utrecht University, Utrecht, The Netherlands; 7 Department of Physiological Chemistry, Centre for Biomedical Genetics and Cancer Genomics Centre, UMC Utrecht, Utrecht, The Netherlands; Duke University Medical Center, United States of America

## Abstract

The endosomal pathway in neuronal dendrites is essential for membrane receptor trafficking and proper synaptic function and plasticity. However, the molecular mechanisms that organize specific endocytic trafficking routes are poorly understood. Here, we identify GRIP-associated protein-1 (GRASP-1) as a neuron-specific effector of Rab4 and key component of the molecular machinery that coordinates recycling endosome maturation in dendrites. We show that GRASP-1 is necessary for AMPA receptor recycling, maintenance of spine morphology, and synaptic plasticity. At the molecular level, GRASP-1 segregates Rab4 from EEA1/Neep21/Rab5-positive early endosomal membranes and coordinates the coupling to Rab11-labelled recycling endosomes by interacting with the endosomal SNARE syntaxin 13. We propose that GRASP-1 connects early and late recycling endosomal compartments by forming a molecular bridge between Rab-specific membrane domains and the endosomal SNARE machinery. The data uncover a new mechanism to achieve specificity and directionality in neuronal membrane receptor trafficking.

## Abstract 2

The neuronal protein GRASP-1 is shown to be a key molecule controlling endosomal trafficking and thereby regulating synapse integrity and synaptic plasticity.

## Author Summary

Neurons communicate with each other through specialized structures called synapses, and proper synapse function is fundamental for information processing and memory storage. The endosomal membrane trafficking pathway is crucial for the structure and function of synapses; however, the components of the neuronal endosomal transport machinery are poorly characterized. In this paper, we report that a protein called GRASP-1 is required for neurotransmitter receptor recycling through endosomes and back to the cell surface, as well as for the normal morphology of dendritic spines—the projections that form synapses—and for synaptic plasticity. We show that GRASP-1 coordinates coupling between early and later steps of the endocytic recycling pathway by binding to Rab4, a regulator of early endosomes, and to another endosomal protein found later in the pathway called syntaxin 13—a so-called SNARE protein involved in membrane fusion. GRASP-1 binds Rab4 with its N terminus and syntaxin 13 with its C terminus, suggesting that these interactions could structurally and functionally link early endosomes to those later in the recycling pathway. We propose a model in which GRASP-1 forms a molecular bridge between different endosomal membranes and the SNARE fusion machinery. Our study thus provides new mechanistic information about endosome function in neurons and highlights GRASP-1 as a key molecule that controls membrane receptor sorting and recycling during synaptic plasticity.

## Introduction

In order to receive, process, and transmit information, neurons need substantially regulated mechanisms to locally redistribute membranes and proteins to synaptic sites. Multiple lines of evidence suggest that the endosomal pathway plays a crucial role in synaptic function and plasticity. At excitatory synapses, the postsynaptic membrane composition is subject to continuous and activity-dependent endocytic cycling of postsynaptic molecules. Based on uptake of extracellular gold particles, visualization of clathrin assembly in living neurons and pre-embedding immunogold electron microscopy, it was shown that endosomal compartments are present in the dendritic shaft and spines and that endocytosis occurs at specialized endocytic zones lateral to the postsynaptic density (PSD) [1]. Using live-cell imaging and serial section electron microscopy, it was demonstrated that recycling endosomes are required for the growth and maintenance of dendritic spines [2]. Membrane recruitment from recycling endosomes is a common mechanism that cells employ to expand the plasma membrane and targets proteins in a polarized manner in such distinct processes as cytokinesis, cell-cell adhesion, phagocytosis, and cell fate determination [3],[4].

Perhaps the strongest evidence for the importance of endocytic recycling in synaptic function originates from the analysis of alpha-amino-3-hydroxy-5-methyl-4-isoxazolepropionic acid (AMPA)-type glutamate receptor (AMPAR) trafficking [5]–[8]. AMPARs are the major excitatory neurotransmitter receptors in the brain, and redistribution of AMPARs in and out of the synapse has emerged as an important mechanism for information storage in the brain [6],[8]. Increased delivery of AMPARs to the postsynaptic membrane leads to long-term potentiation (LTP), whereas net removal of AMPARs by internalization from the surface through endocytosis seems to underlie long-term depression (LTD) [5]–[8]. Like any other internalized membrane protein, endocytosed AMPARs undergo endosomal sorting; they can be degraded in lysosomes or recycled back to the surface membrane [9]–[11]. A popular model holds that the recycling endosomes provides the local intracellular pool of glutamate receptors for LTP [12]. Neuron-enriched endosomal protein of 21 kD (Neep21) and its interacting protein syntaxin 13 are endosomal proteins implicated in regulating AMPAR trafficking during synaptic plasticity [13]. However, it remains unclear how endocytic receptor sorting and recycling is organized and coordinated in neuronal dendrites.

Multiple proteins identified as regulators of endosomal traffic in non-neuronal cells are also important in neuronal endosomes [3],[14]–[16]. Dendritic spines contain the basic components of the endocytic machinery, postsynaptic receptor endocytosis occurs through a dynamin-dependent pathway, and Rab GTPases and their effectors regulate endosomal traffic [17]–[19]. The classic endosomal Rab proteins, Rab5, Rab4, and Rab11, have all been implicated in endosomal receptor and membrane trafficking in dendrites [12],[19]–[23]. Rab5 controls transport to early endosomes (also called sorting endosomes), whereas Rab4 and Rab11 are involved in the regulation of endosomal recycling back to the plasma membrane [24]. The endosomal pathway can be considered as a mosaic of discrete but overlapping domains that are generated and controlled by Rab proteins and their interacting effector protein networks. The communication and transport between sequentially organized Rab domains is thought to be mediated via proteins that are “shared” by both domains. Bivalent effectors, such as Rabenosyn-5 and Rabaptin-5, have been found that connect proximal Rab5 and distal Rab4 domains on early endosomes [25],[26]. However, how Rab4 and Rab11 recycling endosomal domains are coupled is poorly understood.

To gain a better mechanistic understanding of endosome recycling in neurons, we searched for neuronal interacting partners of Rab4 [27]. Using a pull-down and mass spectrometry approach, we identified GRASP-1 as a neuron-specific effector of Rab4 and key component of endocytic recycling in dendrites. GRASP-1 was originally found to interact with glutamate receptor interacting protein (GRIP) and shown to be involved in regulating AMPAR distribution [28]. We show that GRASP-1 is necessary for AMPAR recycling and synaptic plasticity, essential for maintenance of spine morphology and important for endosomal trafficking. GRASP-1 segregates Rab4 from EEA1/Neep21/Rab5-positive early endosomal membranes and coordinates the coupling to Rab11-labelled recycling endosomes via the interaction with t-SNARE syntaxin 13. These results describe a molecular mechanism for regulating recycling endocytosis by GRASP-1.

## Results

### GRASP-1 Is a Rab4-GTP-Binding Protein

**Figure 1:**
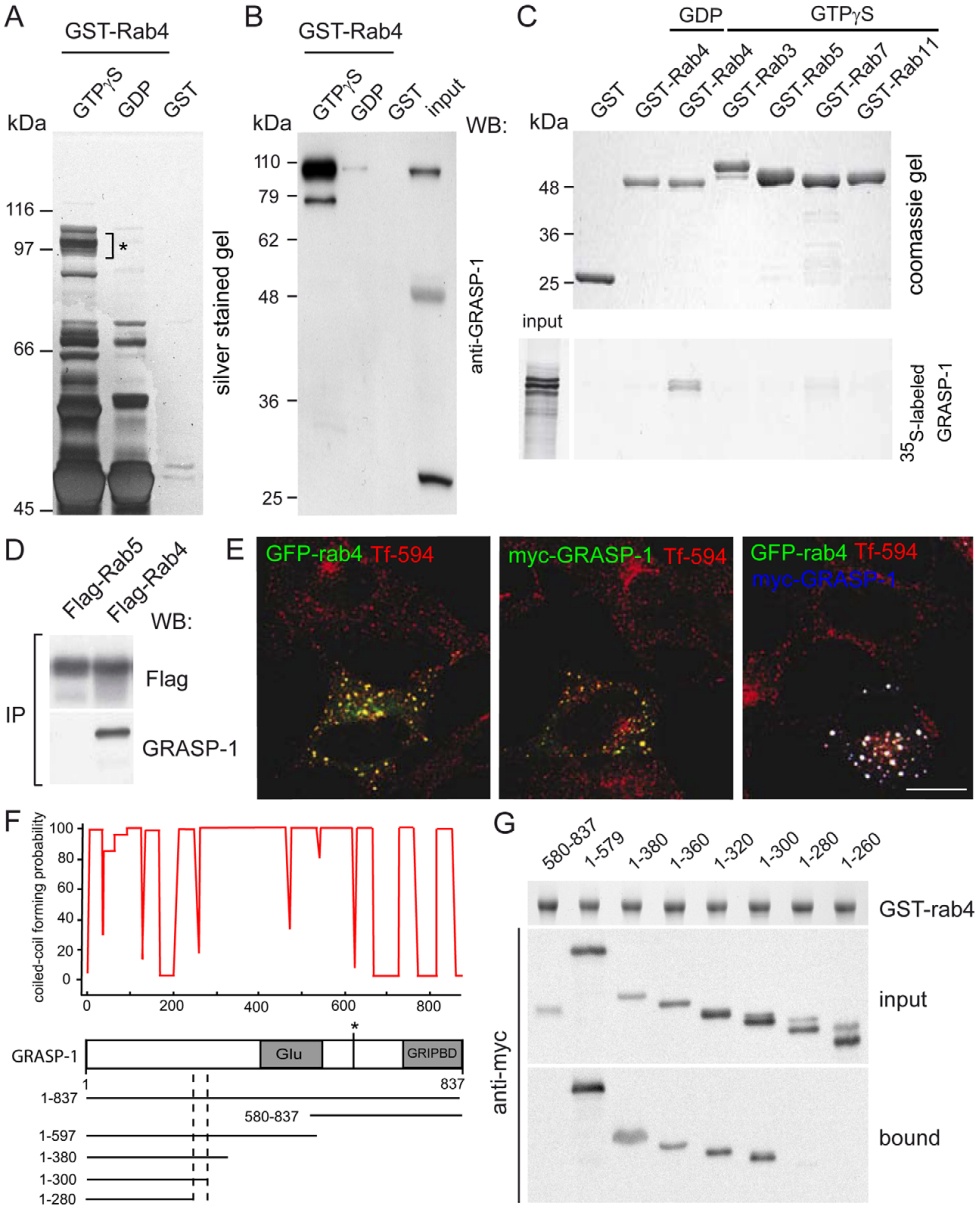
GRASP-1 is a Rab4GTP-binding protein. (A) Silver stained gel showing isolation of GSTRab4-GTPγS binding proteins from brain cytosol. Asterisk denotes band from which GRASP-1 was identified. (B) Western blot of samples from (A) probed with GRASP-1 antibody. (C) Binding assay of ^35^S-labeled GRASP and GSTRab4-GTPγS, or GSTRab4-GDP, and other GTPγS charged GST-Rab proteins. (D) FLAG-tagged Rabs were co-expressed with myc-GRASP-1 in COS-7 cells. Anti-FLAG immunoprecipitates (IP) were analyzed by Western blot with myc antibody. (E) Hela cells were transfected with GFP-Rab4, myc-GRASP-1, or both. Prior to fixation, cells were incubated for 60 min with Alexa594-labeled Tf at 37°C. Bar is 10 µm. (F) Coiled-coil prediction and domain architecture of GRASP-1. Glu, glutamic acid rich domain; asterisk, caspase-3 cleavage site; GRIPBD, GRIP1 binding domain. (G) Binding domain analysis using lysates of COS-7 cells expressing myc-tagged GRASP-1 truncations and GTPγS-charged GST-Rab4.

To identify Rab4-interacting proteins, we performed glutathione S-transferase (GST) pull-down assays with pig brain extracts using GTPγS-loaded GST-Rab4 affinity columns and analyzed the isolated proteins by mass spectrometry (Figure 1A). Among the proteins that were highly enriched in the GST-Rab4-GTPγS pull-downs but were not detected by mass spectrometry in the pull-down assays using GST-Rab4-GDP or GST alone, we found known binding partners of Rab4, such as the bivalent Rab effectors Rabaptin-5 and Rabenosyn-5 (Table 1) [25],[29]. The most significant novel hit was GRASP-1, which was originally identified as a GRIP/AMPAR interacting protein. GRASP-1 has been shown to regulate AMPAR targeting and Jun-N-terminal kinase (JNK) signaling [28],[30]. The association between GRASP-1 and Rab4 was confirmed by immunoblotting with an antibody against GRASP-1 (Figure 1B). Binding of GRASP-1 to Rab4 was direct and specific since GRASP-1 associates with GST-Rab4 but not with the other tested Rab proteins, such as Rab3, Rab5, and Rab11 (Figure 1C). Some weaker binding was detected with Rab7 in this assay. Immunoprecipitation experiments from COS-7 cells co-expressing myc-GRASP-1 and Flag-Rab4 or Flag-Rab5 further confirmed the interaction of GRASP-1 with Rab4 (Figure 1D). Fluorescence microscopic analysis of Hela cells transfected with myc-GRASP-1 and GFP-Rab4 showed that the distribution of GRASP-1 fully coincided with GFP-Rab4 (Figure 1E). Analysis of the endosomal compartment in the same cells, as visualized by internalized Alexa594-Transferrin (Tf-594), indicated that GRASP-1 localizes to the Rab4-positive domain of the early endosomal recycling system. These immunofluorescence data are in line with the reported endosomal localization of GRASP-1 in Hep-2 cells, detected with an autoimmune GRASP-1 serum from a patient with recurrent infections and a presumed immune deficiency [31].

**Table 1:** Binding partners of GST-Rab4-GTP in pig brain extracts identified by mass spectrometry.

Identified Protein	MW (kDa)	Pept. Total	NCBI GI Number	References
Rabaptin-5	99.7	68	1050523	[29]
GRASP-1	96.3	9	16758652	[28]
Rabenosyn-5	89.5	3	58037445	[25]

The table shows proteins identified with a significant Mascot score in GST-Rab4-GTP pull-downs from pig brain extracts. The list is corrected for background proteins, which were identified in a control GST-Rab4-GDP and GST pull-down. For each identified protein, the list is filtered for duplicates and shows only the hits with identified peptides.

GRASP-1 has an extensive propensity to form coiled-coils and contains a caspase-3 cleavage site, a PDZ-like GRIP binding domain, and a central glutamate-rich stretch (Figure 1F). To define the minimal Rab4 binding domain on GRASP-1, we generated a series of myc-GRASP-1 truncations (Figure 1F). GST-Rab4 pull-down assays with COS-7 cell extracts expressing GRASP-1 mutants showed that the N-terminal domain of GRASP-1 binds to Rab4 and that the coiled-coil region between amino acid 280–300 is required for this interaction (Figure 1G). However, full-length GRASP-1 lacking amino acid 280–300 partially retained Rab4 binding (unpublished data). These data argue for an important role of the N-terminal coiled-coil region in Rab4 binding but show that other regions might also be involved.

**Figure 2:**
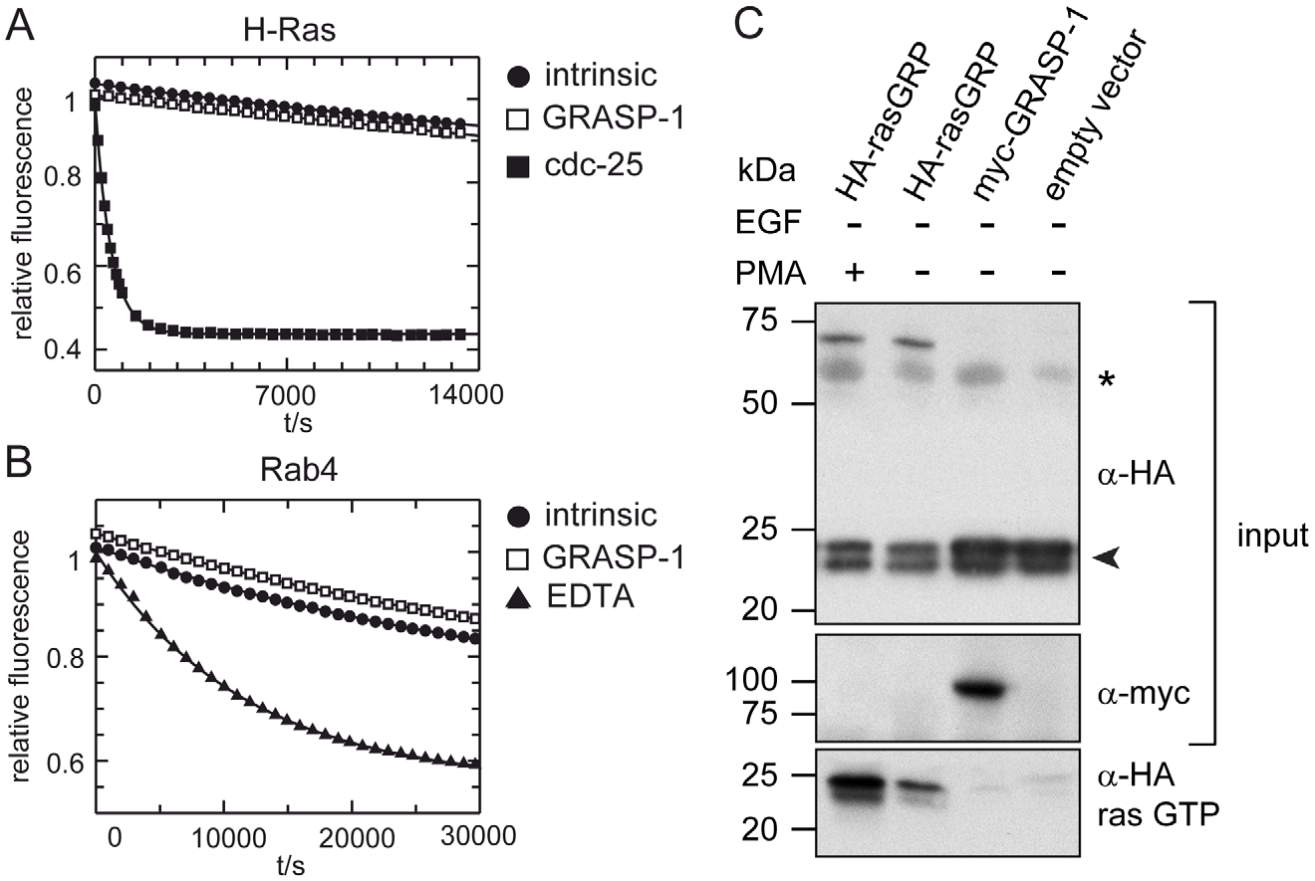
GRASP-1 does not have GEF activity on H-ras and Rab4. (A–B) 0.2 µM H-ras or Rab4 loaded with fluorescent mantGDP was incubated with an excess of GDP at 25°C, in the absence or in the presence of 10 µM GRASP-1(1–594), 0.2 µM cdc-25, or 10 µM EDTA. Dissociation of mGDP was monitored by measuring the decrease in relative fluorescence that accompanies release of mGDP from the GTPase. (C) COS-7 cells were transfected with HA-Hras in combination with indicated constructs and treated with or without PMA. Ras-GTP was isolated on GSH beads containing the ras binding domain of the ras effector raf and analyzed by Western blot with HA antibody. Note that full-length GRASP-1 did not increase rasGTP level above non-transfected control. Asterisk and arrowhead in HA Western blot of input material denote a background band and the position of HA-ras, respectively.

It has been reported that GRASP-1 may serve as a guanine nucleotide exchange factor (GEF) for H-ras [28]. We tested whether GRASP-1 might be a GEF for Rab4 by analyzing recombinant GRASP-1(1–594) in a GEF assay using fluorescent mantGDP. GRASP-1 did not act as GEF for Rab4 (Figure 2A,B). However, unlike the positive control cdc25, GRASP-1 also did not exhibit noticeable GEF activity towards H-ras (Figure 2A). Full-length GRASP-1 also failed to increase GTP-loading of H-Ras in vivo as measured in pull-down assays with the recombinant ras binding domain of Raf-1. The bona fide GEF Ras-GRP markedly increased the amount of H-Ras in the GTP state (Figure 2C), which was further enhanced through its membrane recruitment via a phorbol myristate acetate (PMA)-controlled pathway [32]. In line with these results, careful sequence analysis of GRASP-1 did not reveal significant homology to any known rasGEF. Together these data suggest that GRASP-1 is not a rasGEF but a Rab4 effector.

### GRASP-1 Localizes to a Sub-Domain of Rab4-Positive Early Recycling Endosomes in Neurons

**Figure 3:**
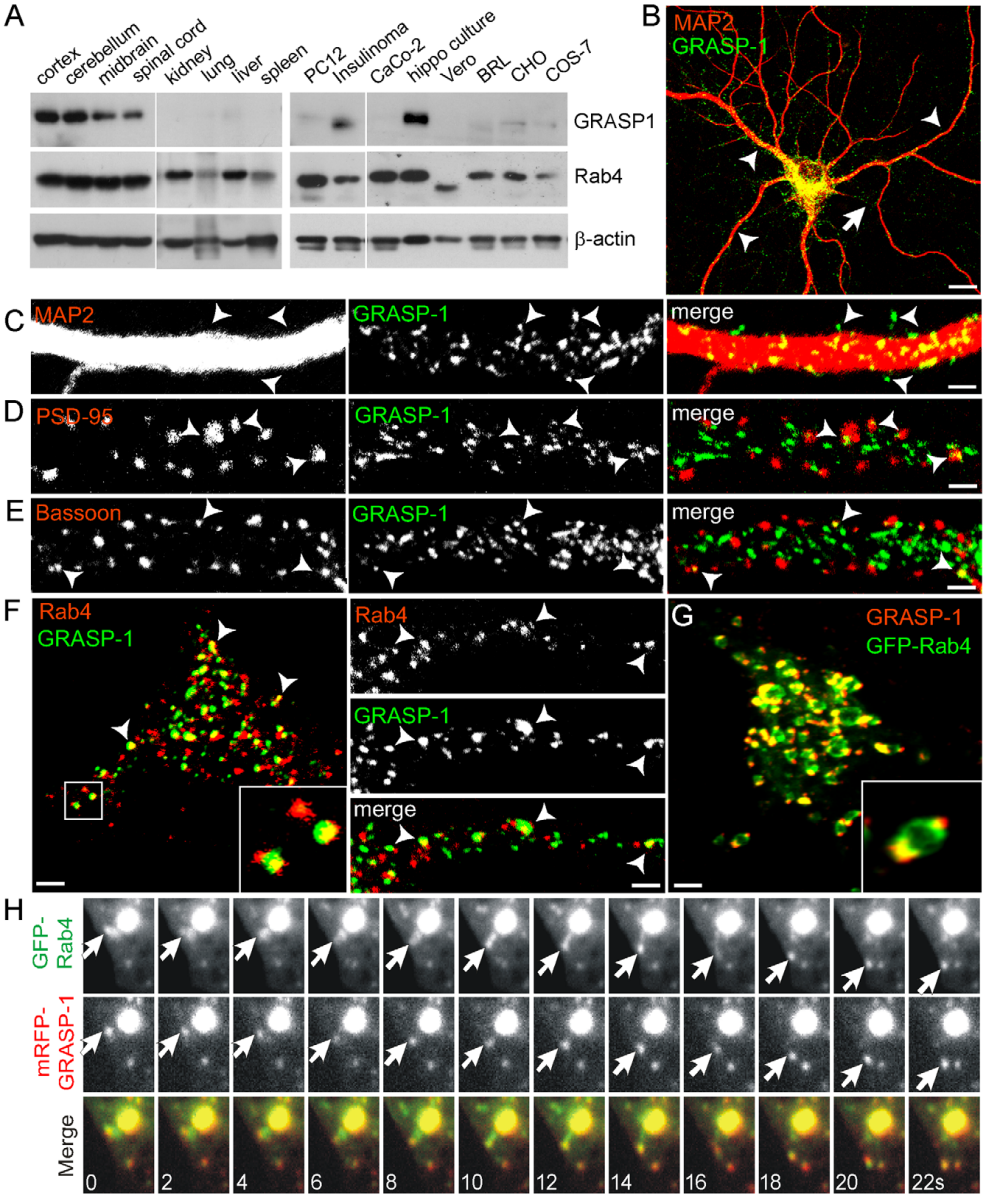
Colocalization of GRASP-1 and Rab4 in hippocampal neurons. (A) Expression pattern of Rab4 and GRASP-1 in mouse tissue and cultured cells visualized on Western blot. (B–F) Representative images of hippocampal neurons double-labeled with antibodies against GRASP-1 and endogenous markers. (B) MAP2 and GRASP-1, arrow denotes axon and arrowheads dendrites. (C) MAP2 and GRASP-1, arrow heads mark GRASP-1 signal beyond the dendritic shaft. (D) PSD-95 and GRASP-1. (E) Bassoon and GRASP-1, arrowheads denote localization of GRASP-1 to synaptic sites. ∼15% of the synapses colocalize with GRASP-1, while the “random” colocalization is ∼5% as determined by rotating the red channel image. (F) Rab4 and GRASP-1 in the cell body (left) and dendrites (right). Arrowheads denote areas of colocalization, inset show magnified regions. Bar in B is 10 µm; Bar in (C–F) is 1 µm. (G) Image of the cell body of hippocampal neurons transfected at DIV13 with GFP-Rab4 and stained for GRASP-1. Magnified region is shown as inset; note the partial localization of GRASP-1 on the distal domain of GFP-Rab4 endosomes. Bar is 1 µm. (H) Simultaneous imaging of GFP-Rab4 (green) and mRFP-GRASP-1 (red) in transfected hippocampal neurons. Successive frames are shown and time (seconds) is indicated in the merge panel.

We examined GRASP-1 expression in mouse tissues and cell lines and showed by Western blot that GRASP-1 is highly expressed throughout the central nervous system, including cortex, cerebellum, midbrain, and spinal cord, and in primary cultured hippocampal neurons but is absent in non-neuronal tissues and cell types with the exception of neuroendocrine insulinoma cells (Figure 3A). These results are consistent with previous immunoblot and immunohistochemistry analyses [28], indicating that GRASP-1 is expressed in neurons throughout the CNS, with highest expression levels in the hippocampus. Double labeling confocal immunofluorescence on mouse brain and spinal cord sections showed that GRASP-1 immunoreactivity was associated with punctate structures throughout the somato-dendritic compartment of neurons (Figure S1 and unpublished data). These punctate structures generally were immunoreactive for Rab4, although various GRASP-1 positive structures did not label for Rab4 and vise versa (Figure S1).

**Figure 4:**
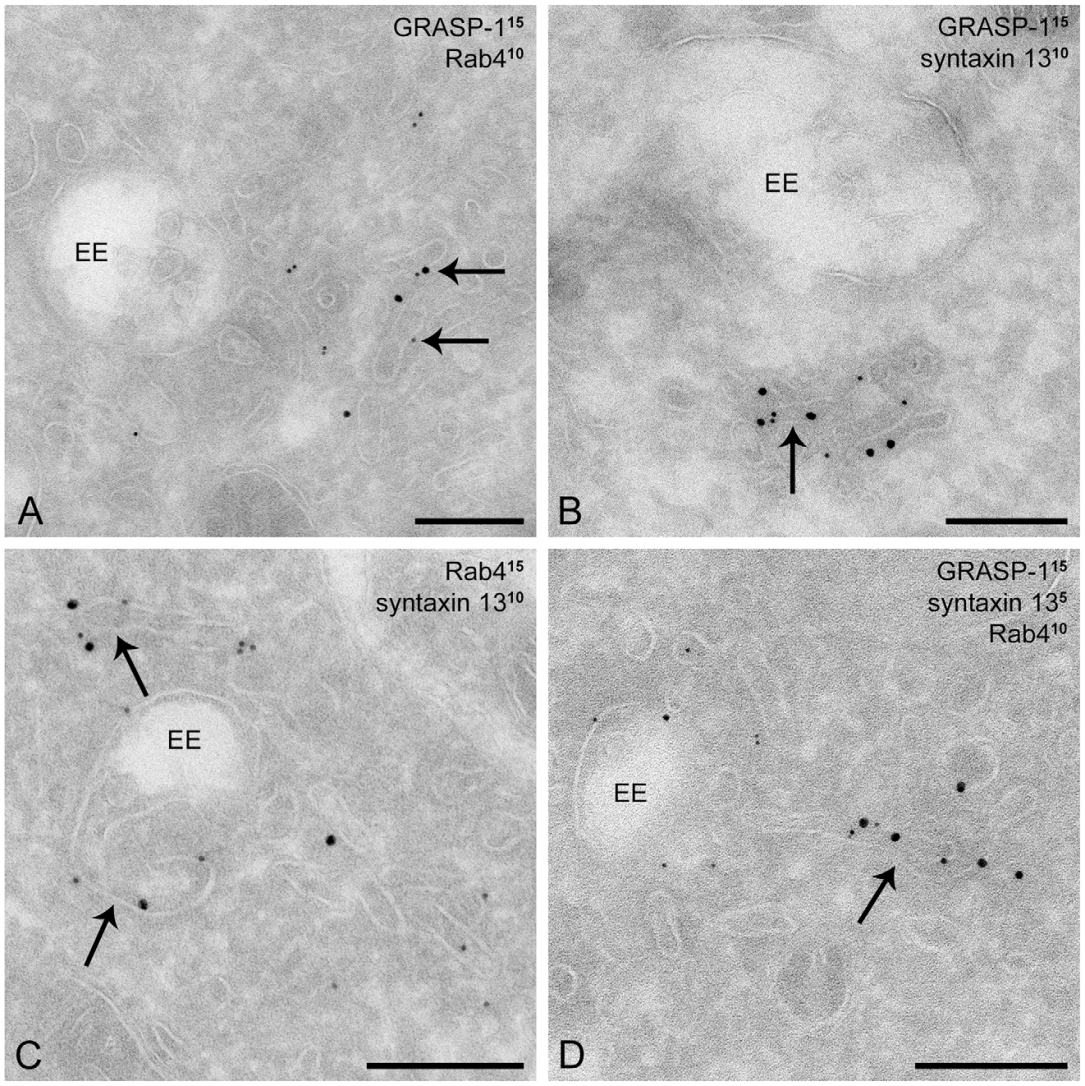
Endogenous GRASP-1, Rab4, and syntaxin 13 coincide on recycling endosomal tubules. Immunogold EM of hippocampal neurons labeled with 10 nm protein A gold for Rab4 and with 15 nm protein A gold for GRASP-1 (A), with 10 nm protein A gold for syntaxin 13 and with 15 nm protein A gold for GRASP-1 (B), with 10 nm protein A gold for syntaxin 13 and with 15 nm protein A gold for Rab4 (C), or with 15 nm protein A gold for GRASP-1, with 5 nm protein gold for syntaxin 13, and with 10 nm protein A gold for rab4 (D). Arrow denotes tubular endosomal membrane to which GRASP-1, syntaxin 13, and Rab4 localized. EE indicates early endosomes and scale bar is 100 nm.

Immunofluorescence labeling in mature hippocampal neurons (>days in vitro 17; DIV 17) revealed that endogenous GRASP-1, although present in axons, is predominantly localized within the somatodendritic compartment, as evidenced by its labeling pattern and the codistribution with the dendritic marker MAP2 (Figure 3B). GRASP-1 is associated with punctate structures that occasionally extend beyond the dendritic shaft (arrowheads in Figure 3C), overlap with the synaptic markers PSD-95 (arrowheads in Figure 3D) and Bassoon (arrowheads in Figure 3E), and localize within the dendritic spines visualized in β-galactosidase (β-gal) filled neurons (unpublished data). In line with the immunohistochemistry data (Figure S1) [28], colocalization of endogenous Rab4 and GRASP-1 is observed in primary hippocampal neurons (Figure 3F). Immunoelectron microscopy showed that endogenous GRASP-1 and Rab4 localize on an extensive tubular network that appeared to emanate from endosomes with a morphology that is characteristic of recycling tubules (Figure 4A). The ability of GRASP-1 to associate with Rab4 positive endosomes was further confirmed by simultaneous dual color live imaging of mRFP-GRASP-1 and GFP-Rab4: GRASP-1 was observed on mobile Rab4-positive vesicles and tubular structures which dock and fuse with larger GRASP-1/Rab4 endosomal domains (Figure 3H; Videos S1 and S2). Overexpression of GFP-Rab4 in hippocampal neurons increased the size of the endosomal structures where GRASP-1 and Rab4 coincide (Figure 3G). Close inspection of these structures revealed that endogenous GRASP-1 localizes to a sub-domain of the large Rab4-positive endosome (Figure 3G, inset), suggesting that GRASP-1 might regulate a particular step in the endosomal recycling pathway. To test whether endosomal GRASP-1 localization depends on Rab4 activity, neurons were transfected with dominant negative Rab4 (Rab4S22N). Expression of Rab4S22N redistributed GRASP-1 away from punctate endosomes, while other endosomal proteins were unaffected (Figure S2). Although it is likely that Rab4S22N inhibits membrane localization of its effector GRASP-1, we cannot exclude that overall levels of GRASP1 are also affected by Rab4S22N. Together these data indicate that GRASP-1 is selectively expressed in neurons, where it is partially localized to Rab4-positive endosomes in dendrites and present in spines near postsynaptic structures.

### GRASP-1 Is Required for Dendritic Spine Morphology

To explore the function of GRASP-1, we used RNA interference to knock down expression of GRASP-1 in mature hippocampal neurons. We found two independent GRASP-1-shRNA sequences (#2 and #5) that specifically inhibited expression of GRASP-1 in hippocampal neurons (Figure S3). GRASP-1 antibodies detected more than ∼80% reduction of GRASP-1 staining intensity in the cell body as well as in dendrites in GRASP-1-shRNA transfected neurons (Figure S3B), while other antibody staining, such as of MAP2, were unaffected (unpublished data). Both GRASP-1-shRNAs constructs produced similar phenotypic effects.

**Figure 5:**
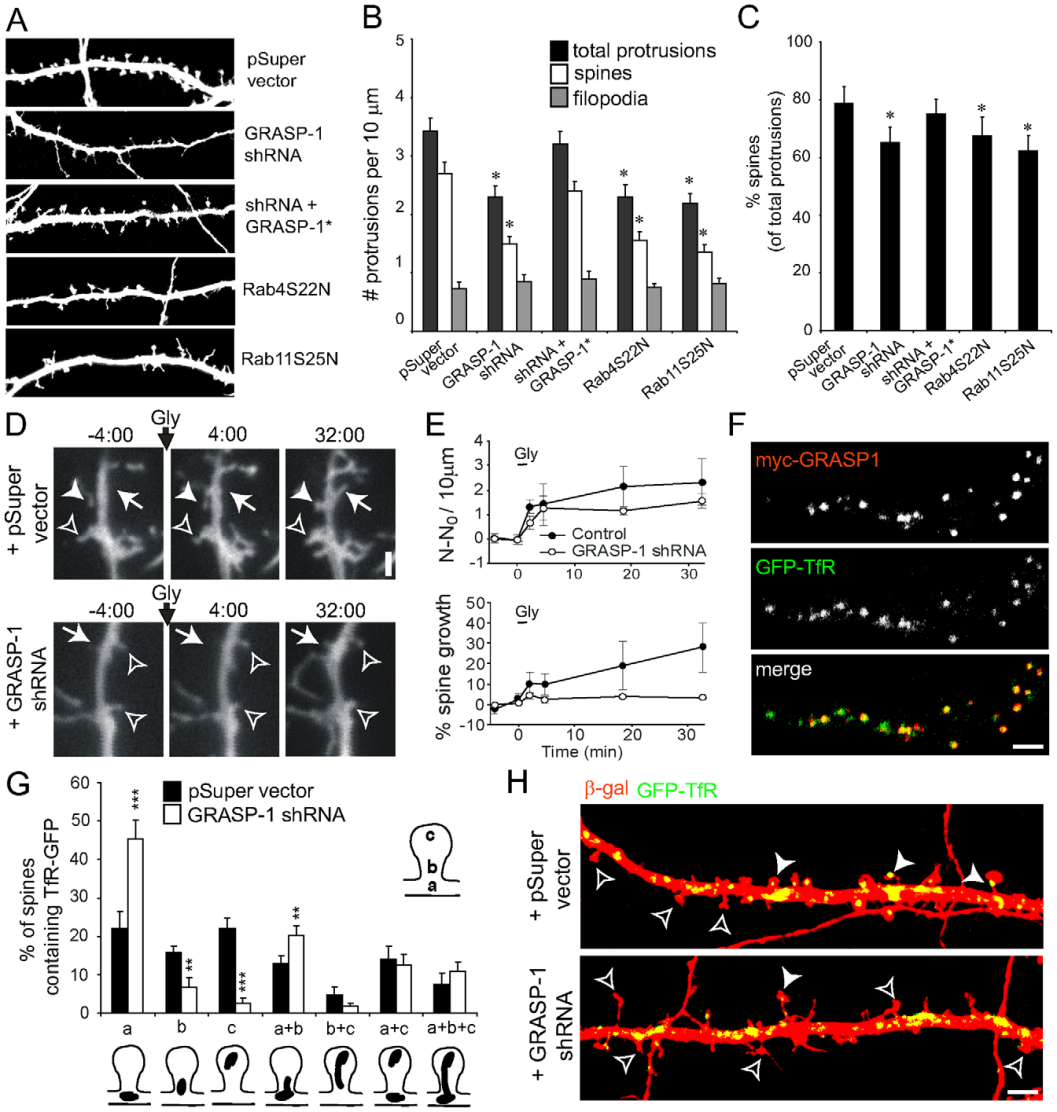
GRASP-1 is required for the maintenance of dendritic spines. (A) Representative high magnification images of dendrites of hippocampal neurons co-transfected at DIV13 for 4 d with β-galactosidase (to mark the dendrites), and either pSuper, pSuper-GRASP-1-shRNA#2, GRASP-1-shRNA#2 and GFP-GRASP-1*, Rab4S22N or Rab11S25N, and labeled with anti-β-galactosidase. (B) Quantification of number of protrusions per 10 µm dendrites in hippocampal neurons transfected as indicated in (A). (C) Percentage of spines of hippocampal neurons transfected as indicated in (A). (D) Neurons expressing GFP (to mark the dendrite), and either pSuper or pSuper-GRASP-1-shRNA#2 were stimulated with glycine (200 mM, 3 min), and then imaged for >30 min after glycine stimulation. Arrows indicated spine formation. Closed and open arrowheads represent spine growth and stable protrusions, respectively. (E) Quantification of protrusion formation (top) and spine growth (bottom) following glycine stimulation. N, number of dendritic protrusions per 10 µm at the indicated time; N_0_, average number of dendritic protrusions per 10 µm before application of glycine. Spine growth was probed as the change in sum of spine widths per 10 µm and comprises both addition of new spines and growth of pre-existing spines. Glycine-stimulated spine growth is blocked by GRASP-1-shRNA#2 (bottom). (F) High magnification images of dendrites of hippocampal neurons cotransfected at DIV13 for 4 d with myc-GRASP-1 (red) and GFP-TfR. (G,H) Percentage of spines containing TfR-GFP positive endosomes at the indicated locations. Hippocampal neurons were co-transfected at DIV13 for 4 d with β-galactosidase (to mark dendrites) and GFP-TfR (to mark endosomes) and pSuper control vector or pSuper-GRASP-1-shRNA#2 as shown in (H). Closed and open arrowheads denote protrusions with and without GFP-TfR marked endosomes in the spine head, respectively. Error bars indicate S.E.M. ** *p*<0.005. *** *p*<0.0005. Bar is 1 µm.

In view of previous observations that inhibition of endosomal recycling by dominant negative forms of Rab4 and Rab11 alters the morphology of dendritic spines [2], we first examined the effect of GRASP-1 knock-down on dendritic spines. In neurons co-expressing GRASP-1-shRNA and β-gal, we observed a marked decrease in the total number of protrusions (Figure 5A). The remaining dendritic protrusions were classified as filopodia-shaped protrusions and mushroom-shaped spines based on the ratio of spine head width to protrusion length. Quantification revealed that knock-down of GRASP-1 decreased the number of mushroom-headed spines (Figure 5B,C). Neurons expressing GRASP-1* (which is resistant to GRASP-1-shRNA#2 knock-down) largely reversed the spine phenotype (Figure 5A–C). A similar spine phenotype was observed by expressing dominant negative forms of Rab11 (Rab11S25N) and Rab4 (Rab4S22N) (Figure 5B,C). We next tested whether GRASP-1 knock-down could inhibit LTP-induced spine growth by glycine stimulation, a protocol used to induce chemical LTP in cultured hippocampal neurons [2]. In control neurons, glycine treatment induced new spine formation and preexisting spine growth, while in the absence of GRASP-1 spine growth is blocked (Figure 5D,E). Together these data indicate that GRASP-1 plays an essential role within the recycling endosomal pathway to maintain dendritic spine morphology and regulate LTP-induced spine growth.

### GRASP-1 Regulates Recycling Endosome Distribution

To directly examine the effect of GRASP-1 knock-down on recycling endosomes distribution in spines, we analyzed its localization with GFP-tagged transferrin receptor (GFP-TfR), which is an archetype recycling cargo that at steady state resides in recycling endosomes [2]. As expected GRASP-1 and GFP-TfR showed a strong colocalisation within dendrites (Figure 5F). TfR-GFP-labeled endosomes were present in the dendritic shaft at the base of spines (a), in the spine neck (b), and in the spine head (c) (Figure 5G). In neurons transfected with GRASP-1-shRNA, GFP-TfR-labeled endosomes were abundantly present in the dendritic shaft at the base of spines but were depleted from the spines (Figure 5H). Quantitative analysis revealed that in control neurons ∼50% of the spines had TfR-GFP-labeled endosomes in their neck and head (b, c, and b+c), whereas in the absence of GRASP-1 only ∼10% of the spines contained recycling endosomes (Figure 5G). These data show that GRASP-1 regulates recycling endosomal localization into dendritic spines and most likely explains the observed GRASP-1 knock-down spine phenotype.

### GRASP-1 Regulates AMPAR Recycling

**Figure 6:**
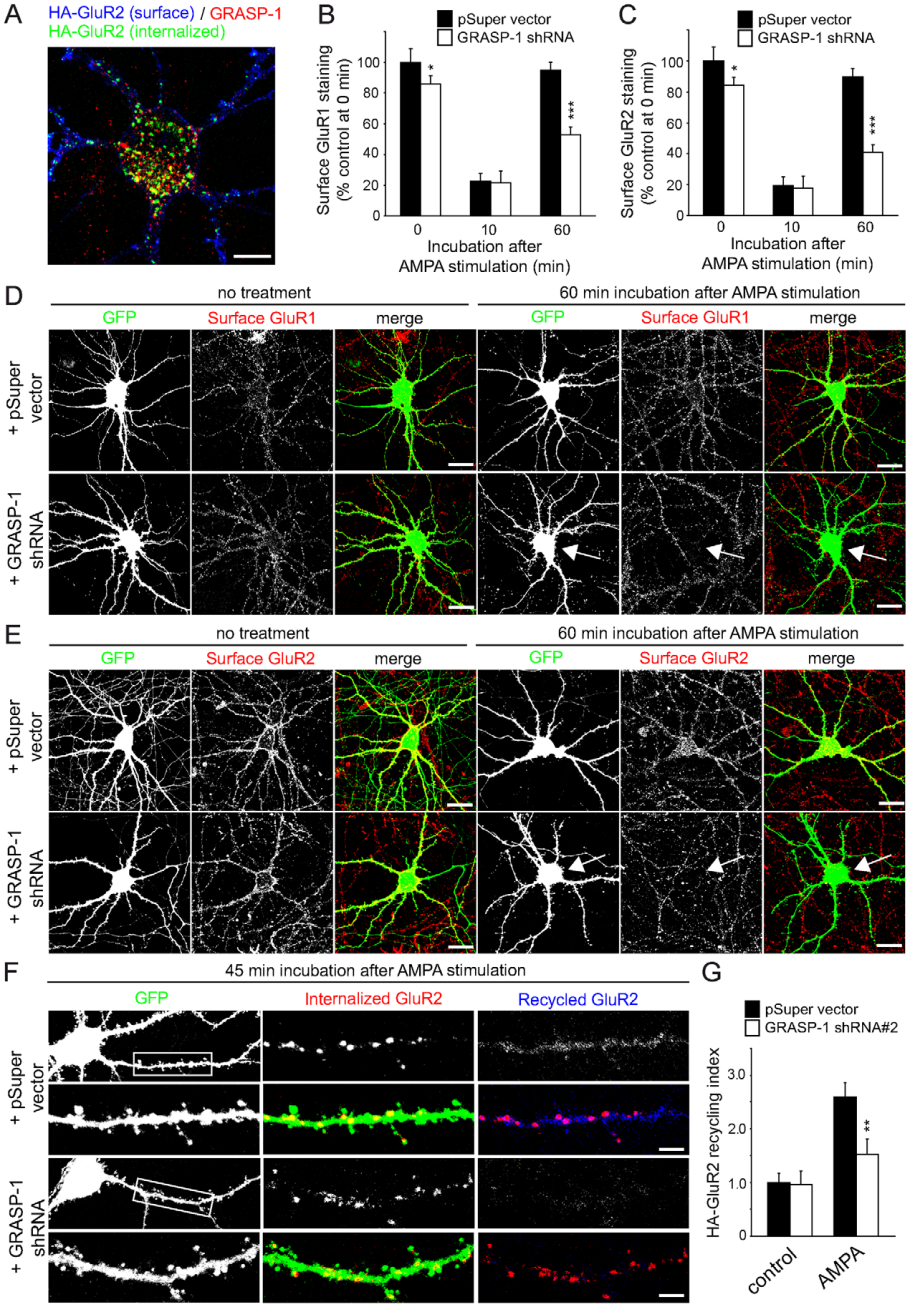
Knock-down of GRASP-1 reduces AMPAR recycling. (A) Representative merge image of surface HA-GluR2 (blue) and internalized HA-GluR2 (green) in soma and dendrites of hippocampal neurons labeled for GRASP-1 (red) after 10 min AMPA stimulation. Bar is 10 µm. (B,C) Quantification of the surface fluorescence intensities of endogenous GluR1 (B) and GluR2 (C) in control pSuper vector or GRASP-1-shRNA#2 transfected neurons. The cells were untreated (0 min) or stimulated with AMPA for indicated times. Histograms show fluorescent intensity of surface GluR subunit staining relative to the intensity of GFP transfected control neurons at basal levels. *n* = 20 cells for each group. (D,E) Representative images of hippocampal neurons stained for endogenous surface GluR1 (D) and GluR2 (E). Hippocampal neurons at DIV13 were cotransfected with GFP and pSuper control vector or GRASP-1-shRNA#2. At DIV17, neurons were fixed (0 min, no treatment) or stimulated for 2 min with 100 µM AMPA in the presence of 50 µM APV and further incubated for a total of 10 or 60 min before fixation. Endogenous surface GluR1 (D) or GluR2 (E) was revealed by immunofluorescence labeling without permeabilization using specific extracellular AMPAR antibodies. Bar is 20 µm. (F) Neurons transfected with GFP, HA-GluR2, and either pSuper control vector or GRASP-1-shRNA#2 were stained live with an anti-HA antibody, stimulated for 2 min with AMPA/APV, acid stripped, and incubated in conditioned media for 45 min. Recycled HA-GluR2 (blue) and internalized HA-GluR2 (red) were sequentially labeled. Bar is 1 µm. (G) Quantification of the ratio of recycled to internalized HA-GluR2 and normalized to unstimulated wild-type control neurons (HA-GluR2 recycling index) as indicated in (F). Error bars indicate S.E.M. * *p*<0.05. ** *p*<0.005. *** *p*<0.0005.

To further explore the functional importance of GRASP-1 in endosomal recycling, we studied the effect of GRASP-1 knock-down on endocytic trafficking of AMPAR. First, we analyzed GRASP-1 colocalization with internalized AMPARs by using the fluorescence-based antibody feeding assay [10]. Live hippocampal neurons expressing extracellular HA-tagged GluR1 or GluR2 subunits were surface labeled with HA antibody, stimulated with AMPA (100 µM, in the presence of 50 µM APV, a selective n-methyl-D-aspartic acid (NMDA) receptor antagonist), fixed, permeabilized, and stained for internalized GluR subunits and endogenous GRASP-1. At 2 min after AMPA stimulation, only ∼5% of internalized HA-GluR1 or HA-GluR2 colocalized with GRASP-1 (Figure S4A,B). After 10 min following stimulation, colocalization between internalized GluR subunits with GRASP-1 was increased to ∼30% (Figure 6A, Figure S4A,B), which is consistent with the kinetics of internalized AMPAR colocalization with Rab4 [9].

Next, we transfected hippocampal neurons either with GFP and control vector or GFP with GRASP-1-shRNA and analyzed internalization and recycling of endogenous AMPAR following AMPA stimulation by immunolabeling for surface GluR1 and GluR2. At steady state, GRASP-1 knock-down neurons showed a modest but significant reduction (∼15%) in surface labeling for GluR1 (Figure 6B,D) and GluR2 (Figure 6C,E) compared to controls. After 10 min of stimulation, GluR1 and GluR2 decreased at the neuronal surface in both control and GRASP-1 shRNA expressing neurons, reflecting receptor internalization (Figure 6B,C). At 60 min, reappearance of both GluR1 and GluR2 was strongly impaired (∼50%) by GRASP-1 shRNA compared to controls (Figure 6B–E). Consistently, in a protocol where surface HA-GluR2 receptors were stripped away after labeling [33], recycling of HA-GluR2 back to the surface was significantly decreased in neurons expressing GRASP-1-shRNA compared to control neurons (Figure 6F). No difference was observed in the level of intracellular HA-GluR2 after 8 min AMPA stimulation (Figure S4C,D). However, we observed that in GRASP-1 knock-down neurons, more intracellular HA-GluR2 is present in LAMP-1 positive lysosomal compartments after AMPA treatment (Figure S4E,F). These data show that GRASP-1 is important for activity-induced AMPAR recycling.

### GRASP-1 Regulates Synaptic Plasticity

**Figure 7:**
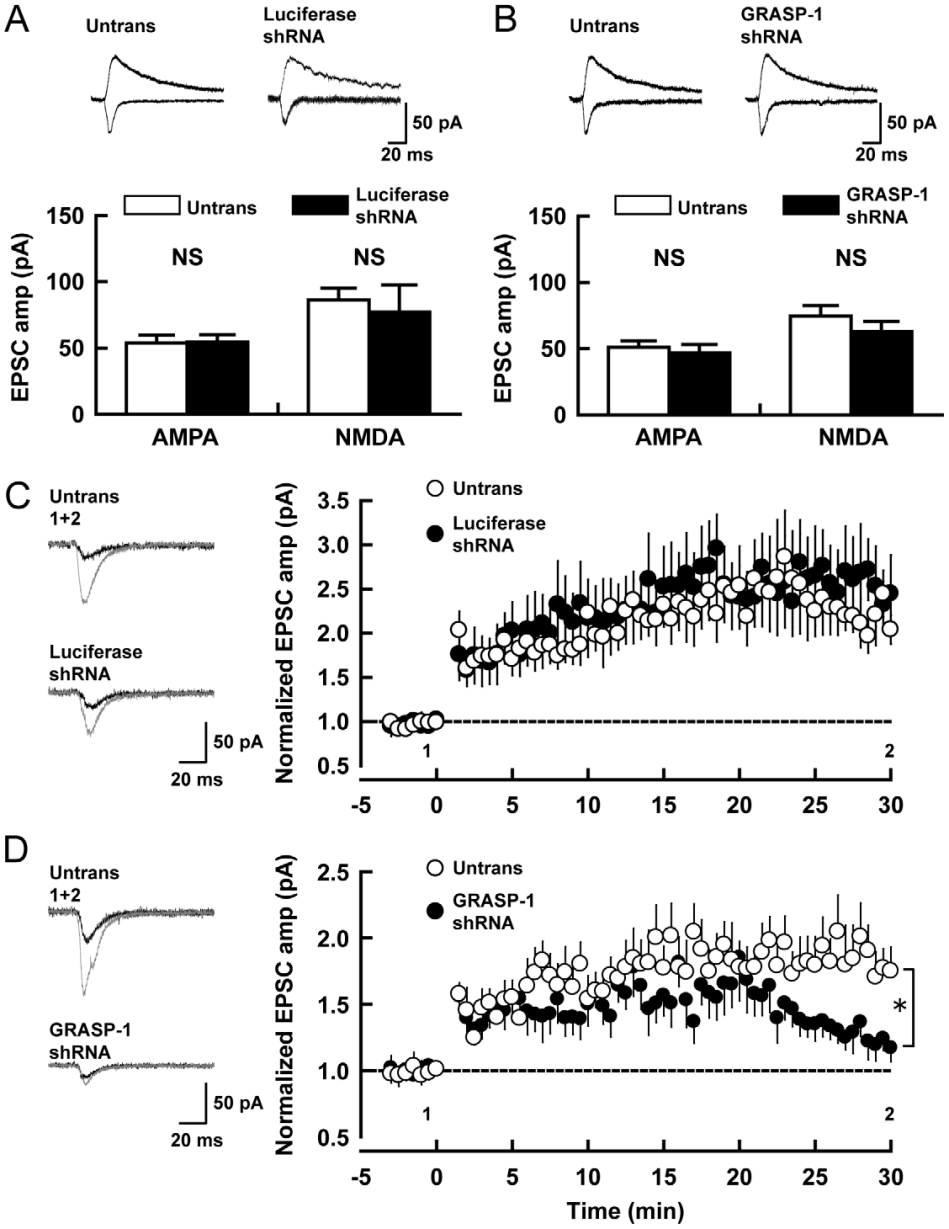
Effect of GRASP-1 knock-down on synaptic transmission and plasticity in hippocampal slice. (A,B) AMPA and NMDA receptor-mediated excitatory synaptic responses were measured from neurons transfected with Luciferase-shRNA (A, control) and GRASP-1-shRNA#5 (B). Top, sample traces mediated by AMPAR (downward) and NMDAR (upward) from pairs of shRNA transfected (Luciferase or GRASP-1-shRNA#5) and neighboring untransfected (Untrans) neurons. Stimulus artifacts were truncated from the traces. Bottom, summary graphs of EPSC amplitudes (AMPA-R-EPSCs and NMDA-R-EPSCs) from shRNA transfected and neighboring untransfected cells. Number of cell pairs: Luciferase-shRNA, 18 and 10; GRASP-1-shRNA#5, 15 and 8 for AMPA and NMDAR-EPSC. NS, not significant. Error bars indicate S.E.M. (C,D) LTP was induced in shRNAs expressing and neighboring untransfected cells by pairing depolarization to 0 mV with 2 Hz stimulation for 100s. Left, sample AMPAR-EPSC traces from untransfected and Luciferase or GRASP-1 shRNA transfected neurons. Currents before (black) and after (gray) are superimposed. Right, time course of AMPA-EPSCs after LTP induction (LTP was induced at *t* = 0). The time points at which sample traces were obtained are indicated by 1 and 2. Number of cell pairs: Luciferase-shRNA, 6; GRASP-1-shRNA#5, 8. * *p*<0.05.

Next we examined the role of GRASP-1 in excitatory transmission and LTP and recorded excitatory synaptic responses from CA1 pyramidal neurons in organotypic cultures of hippocampal slices. Simultaneous recordings were obtained from both transfected neurons (identified by cotransfected GFP) and a neighboring untransfected neuron. Both control luciferase-shRNA and GRASP-1-shRNA expressing cells had no effect on basal AMPAR-mediated excitatory postsynaptic currents (EPSCs) (GRASP-1 shRNA#5: 0.93±0.09-fold relative to untransfected cells, luciferase shRNA: 1.21±0.18) and NMDAR-EPSCs (GRASP-1 shRNA#5: 0.86±0.09-fold, luciferase shRNA: 1.03±0.32) (Figure 7A,B). The importance of GRASP-1-mediated AMPAR recycling in slices became more evident by testing for synaptic plasticity. After induction of LTP, cells expressing GRASP-1 shRNA induced comparable levels of potentiation to that of neighboring untransfected cells up to 20 min after the LTP induction protocol. Subsequently, however, the response from GRASP-1 shRNA transfected cells started to fall and eventually returned to the baseline level at 30 min after LTP induction (Figure 7C, untransfected neuron: 1.75±0.18-fold enhancement of EPSC at 29–30 min after LTP induction, transfected neuron: 1.17±0.10). In contrast, control luciferase shRNA transfected, and neighboring untransfected neurons expressed stable LTP lasting for at least 30 min (Figure 7D, untransfected neuron: 2.04±0.16-fold enhancement of EPSC, transfected neuron: 2.45±0.44). These data indicate that GRASP-1 is important for synaptic plasticity and particularly for the phase of LTP after the first 20 min. The results suggest that delivery of AMPAR from recycling endosomes might be important for this later phase of LTP.

### GRASP-1 Segregates Rab4 from EEA1/Neep21 Endosomal Membranes

**Figure 8:**
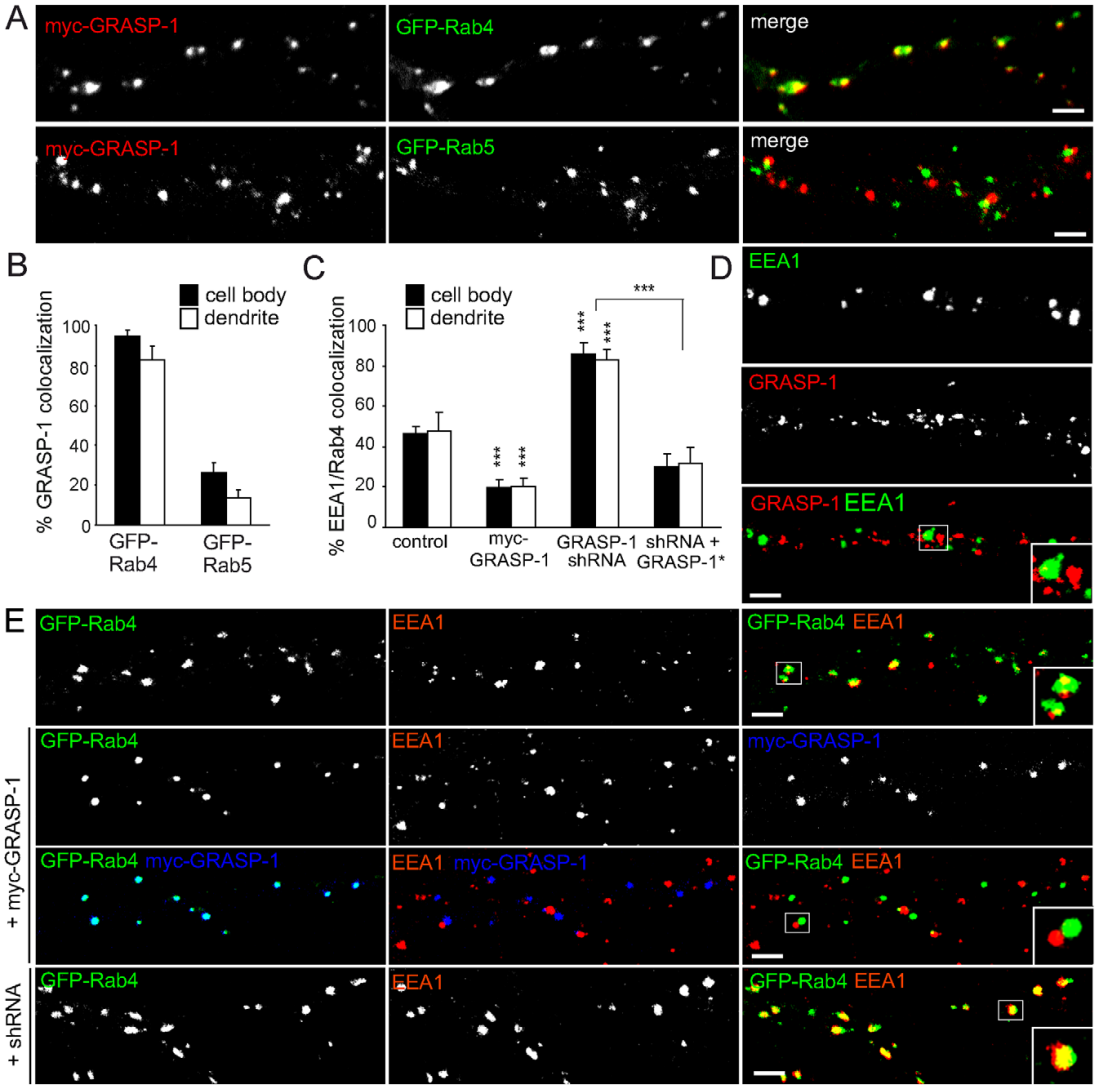
GRASP-1 segregates Rab4 from EEA1 positive endosomal membranes. (A) Representative images of dendrites of hippocampal neurons cotransfected at DIV13 for 4 d with myc-GRASP-1 (red) and either GFP-Rab4 (upper row) or GFP-Rab5 (bottom row). (B) Percentage of colocalization between myc-GRASP-1 and Rab proteins in neurons as indicated in (A). (C) Percentage of Rab4 and EEA1 colocalization in cell body and dendrites as indicated in (E). Error bars indicate S.E.M. *** *p*<0.0005. (D) Representative images of dendrites of hippocampal neurons double-labeled with anti-GRASP-1 (red) and anti-EEA1 (green) antibodies. (E) Representative images of dendrites of hippocampal neurons cotransfected at DIV13 for 4 d with GFP-Rab4 and pSuper control vector, myc-GRASP-1, or pSuper-GRASP-1-shRNA#2 and labeled with anti-EEA1 (red) and anti-myc (blue) antibodies. Bar is 1 µm.

To define more precisely the function of GRASP-1 within the endosomal system, we first examined the localization of exogenous GRASP-1 with respect to early endosomal marker proteins in Hela cells. We found little if any co-distribution with GFP-Rab5 but extensive colocalization with GFP-Rab4 (Figure S5). The same results were obtained in transfected hippocampal neurons, where >80% of Rab4 structures contained GRASP-1 both in dendrites and the cell body, while little overlap was seen with Rab5 (Figures 8A,B, 6). In agreement with this observation, the Rab5 domain marker EEA1 and endogenous GRASP-1 displayed mutually exclusive distributions (Figure 8D), whereas ∼40% of EEA1 structures in the cell body and dendrites colocalized with GFP-Rab4 (Figure 8C,E, top row). These results suggested that Rab4 in neurons is interfaced between a proximal EEA1 and distal GRASP-1 endosomal domain.

To determine whether the endosomal domain organization is regulated by GRASP-1, we knocked down the expression of GRASP-1 and then assayed the co-distribution of EEA1 and GFP-Rab4. Hippocampal neurons transfected with GRASP-1-shRNA showed a strong increase in colocalized EEA1 and GFP-Rab4 (∼80%) compared to control neurons (∼40%) (Figure 8C,E). In contrast, in neurons transfected with myc-GRASP-1, the overlap between EEA1 and GFP-Rab4 was significantly decreased (∼20%) (Figure 8C,E). Similar results were obtained in Hela cells, where myc-GRASP-1 strongly reduced colocalization between GFP-Rab4 and EEA1, while the co-distribution of GFP-Rab5 and EEA1 was not affected (Figure S7). To confirm our results we tested the effect of GRASP-1 on the localization of other early endosomal markers, such as Neep21 [13]. Endogenous Neep21 staining strongly coincides with Rab5 and EEA1 (∼80%) and to a lesser extent with Rab4 (∼40%) (Figure S8 and unpublished data). However in neurons transfected with myc-GRASP-1 the overlap between Neep21 and GFP-Rab4 was significantly reduced (∼20%), consistent with the effect on EEA1 distribution (Figure S8D). In contrast, GRASP-1-shRNA enhances Neep21/Rab4 colocalization (Figure S8D). Together these results suggest that GRASP-1 is able to separate Rab4 from EEA1/Neep21 endosomal domains.

### GRASP-1 Regulates the Coupling between Rab4 and Rab11 Domains

**Figure 9:**
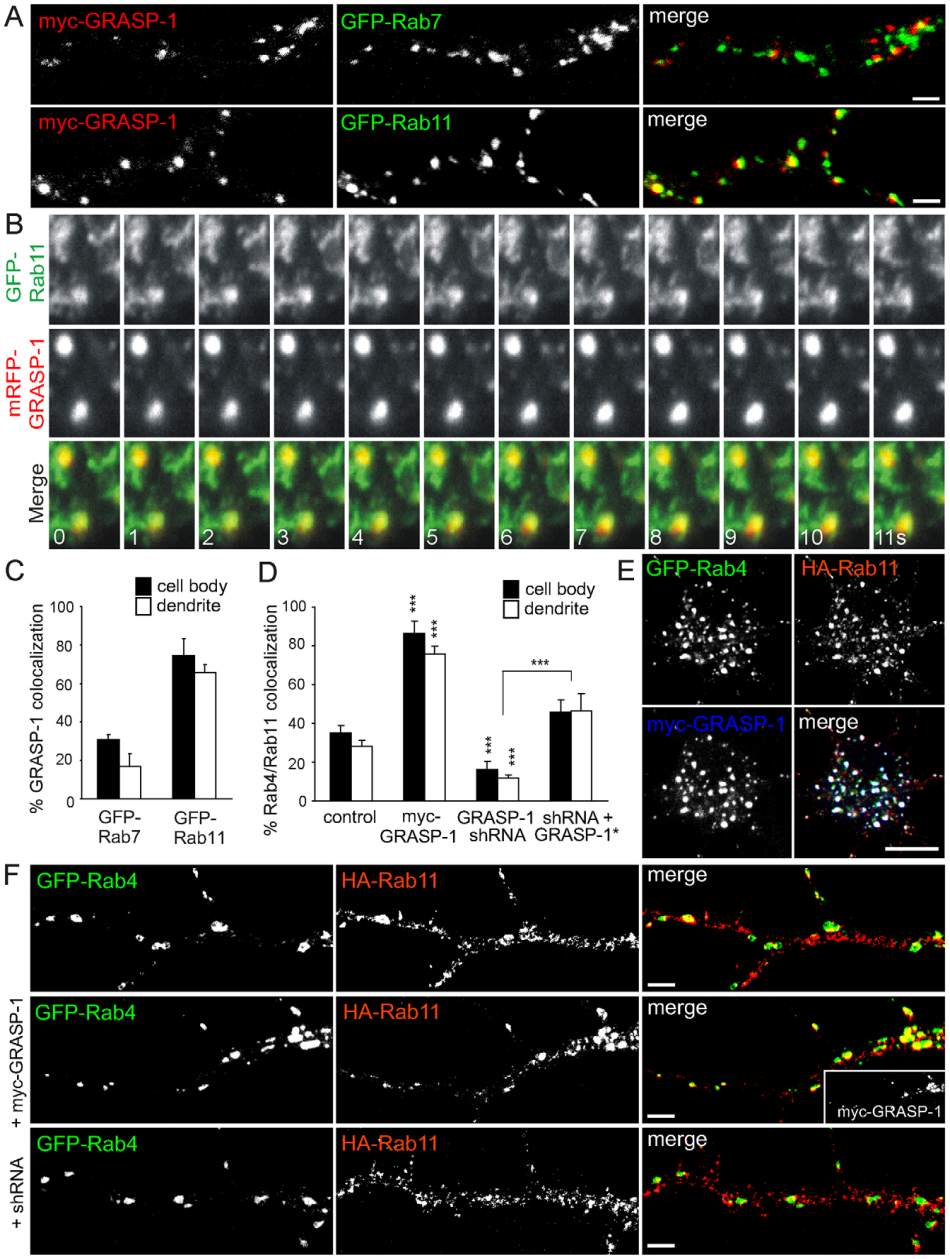
GRASP-1 couples Rab4 and Rab11 domains. (A) Representative images of dendrites of hippocampal neurons cotransfected at DIV13 for 4 d with myc-GRASP-1 and either GFP-tagged Rab7 or Rab11 and labeled with anti-myc (red). Bar is 1 µm. (B) Simultaneous imaging of GFP-Rab11 (green) and mRFP-GRASP-1 (red) in transfected hippocampal neurons. Successive frames are shown and time (seconds) is indicated in the merge panel. (C) Percentage of colocalization between myc-GRASP-1 and Rab proteins in neurons as indicated in (A). Error bars indicate S.E.M. *** *p*<0.0005. (D) Percentage of colocalization between Rab4 and Rab11 domains in neurons co-transfected with GFP-Rab4 and HA-Rab11 with either myc-GRASP-1, pSuper-GRASP-1-shRNA#2, or pSuper-GRASP-1-shRNA#2 and GFP-GRASP-1* as indicated in (F). (E) Images of cell body of hippocampal neurons triple transfected at DIV13 for 4 d with GFP-Rab4, HA-Rab11, and myc-GRASP-1 and labeled with anti-HA (red) or anti-myc (blue) antibodies. Bar is 10 µm. (F) Representative images of dendrites of hippocampal neurons cotransfected at DIV13 for 4 d with GFP-Rab4 and HA-Rab11 and pSuper control vector, myc-GRASP-1, or pSuper-GRASP-1-shRNA#2 and labeled with anti-HA (red) or anti-myc (inset) antibodies. Bar is 1 µm.

We next determined GRASP-1 localization with respect to late and recycling endosomal markers in Hela cells (Figure S6) and hippocampal neurons (Figure 9A). We found little GRASP-1 colocalization with the Rab7 endosomal domains, whereas GRASP-1 labeling coincided extensively with Rab11-positive compartments (∼70%) (Figures 9A,C, S6). These data strongly suggest that GRASP-1 is localized to distal aspects of the endosomal recycling pathway and might serve to couple Rab4 and Rab11 domains. This observation was confirmed by simultaneous dual color live imaging of mRFP-GRASP-1 and GFP-Rab11: GRASP-1 and Rab11 colocalize on larger endosomal domains, while dynamic Rab11-positive structures segregate into distinct tubular or vesicular structures (Figure 9B; Videos S3 and S4). Most motile Rab11-positive tubules only transiently overlap with GRASP-1-positive endosomes (Videos S3 and S4). Rab4, Rab11, and GRASP-1 largely localized to overlapping regions on these large endosomal structures in the neuronal cell bodies and dendrites (Figure 9E). We further explored a possible role for GRASP-1 in coupling Rab4 and Rab11 domains by determining the Rab4/Rab11 co-distribution when GRASP-1 was knocked down as well as after overexpression of myc-GRASP-1. In absence of GRASP-1 we observed a significant decreased Rab4/Rab11 colocalization (15%), compared to control neurons (30% Rab4/Rab11 colocalization), while transfected myc-GRASP-1 robustly enhanced the coalescence of Rab4 and Rab11 domains (80% Rab4/Rab11 colocalization) (Figure 9D,F). Importantly, the observed decrease in EEA1/Rab4 and Neep21/Rab4 domain coupling after myc-GRASP-1 transfection (Figure 8C,E) is consistent with an increase in Rab4/Rab11 domain coupling, while the reverse occurred after GRASP-1 knock-down. These data therefore show that GRASP-1 is a positive regulator of endosomal recycling membrane maturation, via coupling of Rab4- and Rab11-positive endosomal domains.

### Syntaxin 13 Binds to GRASP-1 and Connects Recycling Endosomal Domains

**Figure 10:**
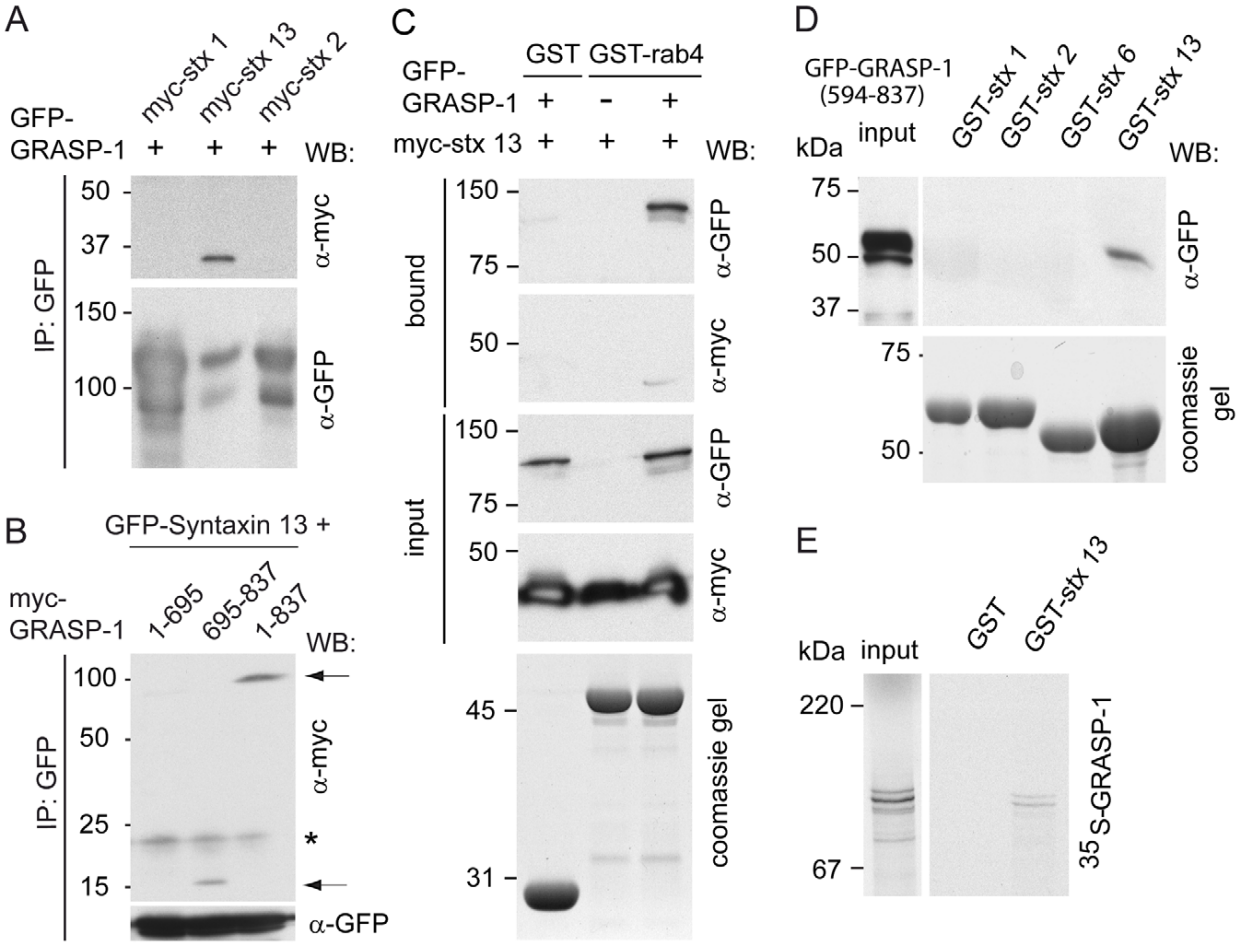
Syntaxin 13 interacts with the C-terminal domain of GRASP-1. (A) Lysates of COS-7 cells cotransfected with GFP-GRASP-1 and myc-syntaxins were immunoprecipitated with anti-GFP antibody and analyzed by Western blot. (B) Lysates of COS-7 cells cotransfected with GFP-syntaxin 13 and full-length myc-GRASP-1 (1–837) or truncated myc-GRASP-1 constructs (1–695 or 695–837) were immunoprecipitated with anti-GFP antibody and analyzed by Western blot. Asterisk indicates background band. Arrows point to co-precipitated GRASP-1 proteins. (C) Binding assay using lysates of COS-7 cells expressing myc-syntaxin 13 with or without GFP-GRASP-1 and GMP-PNP-charged GST-rab4. Note that myc-syntaxin 13 is only isolated on the beads in the presence of GRASP-1. (D) Binding assay using lysate of COS-7 cells transfected with GFP-GRASP-1(594–837) and GST-syntaxins without transmembrane domain (ΔTM). GRASP-1 was analyzed by Western blot with antibody against GFP. (E) Binding assay of ^35^S-labeled GRASP-1 and immobilized GST-syntaxin 13ΔTM.

**Figure 11:**
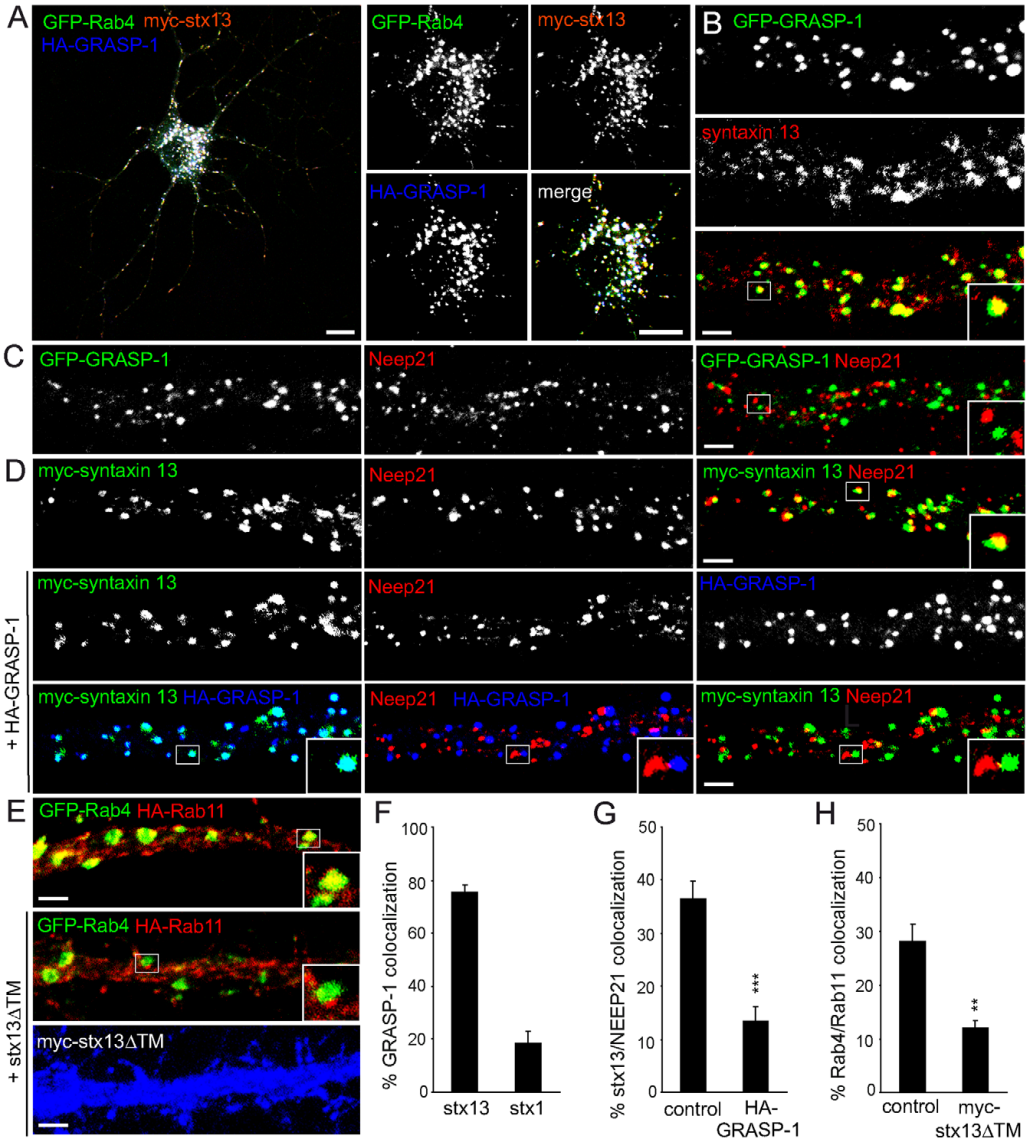
Syntaxin 13 coincides with GRASP-1 and segregates Rab4/Rab11 domains. (A) Representative image of hippocampal neuron triple transfected at DIV13 for 4 d with GFP-Rab4, HA-GRASP-1, and myc-syntaxin 13 and labeled with anti-HA (blue) or anti-myc (red) antibodies. Magnified region of the cell body is shown to indicate the strong colocalization of GRASP-1, Rab4, and syntaxin 13. (B) Representative images of dendrites of hippocampal neurons transfected at DIV13 with GFP-GRASP-1 for 4 d and labeled with anti-syntaxin 13 (red). (C) Representative images of dendrites of hippocampal neurons transfected at DIV13 with GFP-GRASP-1 for 4 d and labeled with anti-Neep21 (red). (D) Representative images of dendrites of hippocampal neurons cotransfected at DIV13 for 4 d with myc-syntaxin 13 and control vector or HA-GRASP-1 and labeled with anti-myc (green), anti-HA (blue), and anti-Neep21 (red). (E) Representative images of dendrites of hippocampal neurons cotransfected at DIV13 for 4 d with GFP-Rab4, HA-Rab11, and control vector or myc-syntaxin 13ΔTM and labeled with anti-myc (blue) and anti-HA (red). (F) Percentage of colocalization between HA-GRASP-1 and myc-syntaxin 1 or myc-syntaxin 13 in neurons. (G) Percentage of colocalization between myc-syntaxin 13 and Neep21 in dendrites as indicated in (D). (H) Percentage of colocalization between GFP-Rab4 and HA-Rab11 domains in dendrites expressing myc-syntaxin 13ΔTM as indicated in (E). Error bars indicate S.E.M. ** *p*<0.005. *** *p*<0.0005. Bar in A is 10 µm; Bar in (B–E) is 1 µm.

GRASP-1 colocalized with endogenous Rab11 (Figure S6) and GFP-Rab11 (Figure 9A) in neurons but did not directly bind to Rab11 (Figure 1C). These observations suggest a crosstalk between GRASP-1 and other proteins on Rab11 endosomal domains in hippocampal neurons. One of these candidate proteins is the SNARE syntaxin 13, a transmembrane domain protein that localizes to Rab11 positive tubular recycling endosomes [34],[35] and is important for AMPAR recycling, spine morphology, and endosomal mobility [2],[12]. We investigated the possible interaction between GRASP-1 and syntaxin 13 by co-immunoprecipitation experiments from COS-7 cells transfected with GFP-GRASP-1 and different myc-syntaxin constructs. GFP-GRASP-1 precipitated syntaxin 13 and not myc-syntaxin 1 and myc-syntaxin 2 (Figure 10A). Consistent, GRASP-1 colocalized with syntaxin 13 (Figures 11A,B and S9) and not with syntaxin 1 (Figure S9A and unpublished data). Moreover, overexpression of GRASP-1 strongly accumulates syntaxin 13 in GRASP-1/Rab4/Rab11 positive structures in neurons (Figure 11A) and Hela cells (Figure S9B). Immunogold EM of neurons showed that syntaxin 13 colocalized with GRASP-1 (Figure 4B) and with Rab4 (Figure 4C) on endosomal tubulovesicular recycling structures, reminiscent of the Rab4-GRASP-1 organelles (Figure 4A). Triple label immuno EM of endogenous Rab4, GRASP-1, and syntaxin 13 indeed revealed partial co-distribution to the endosomal tubulovesicular recycling structures (Figure 4D). This suggests that the three proteins might be engaged in a complex on endosomal membranes. In agreement with this hypothesis, myc-syntaxin 13 could be isolated from COS-7 lysates on GST-Rab4 beads, only if GRASP-1 was co-transfected (Figure 10C). The interaction required the PDZ-like domain containing C-terminal region of GRASP-1, but not the N-terminal Rab4 binding domain (Figure 10B,D), and could be recapitulated with purified GST-syntaxin 13 and ^35^S-labeled GRASP-1 (Figure 10E). Since syntaxin 13 has a transmembrane domain, it could be an anchor for GRASP-1 on endosomal membranes. In accord, the C-terminal part of GRASP-1 is necessary for the localization of GRASP-1 to TfR containing endosomes (Figure S10). However, GRASP-C alone is not sufficient for GRASP-1 membrane localization since the Rab4 binding domain is also required (Figure S10).

Previously, syntaxin 13 was found in complexes with early endosomal proteins EEA1 [36] and Neep21 [13]. To better understand the role of syntaxin 13 in both early and recycling endosomes, we first investigated the distribution of syntaxin 13 in dendrites of hippocampal neurons and found ∼40% overlap between Neep21 and syntaxin 13 (Figure 11D,F), ∼40% colocalization between GRASP-1 and endogenous syntaxin 13 (Figure 11B), while no co-distribution of Neep21 with GRASP-1 was observed (Figure 11C, Figure S6). These data suggest that GRASP-1/syntaxin 13 and Neep21/syntaxin 13 are associated with distinct endosomal structures. Since expression of GFP-GRASP-1 strongly accumulates endogenous syntaxin 13 in the cell body and dendrites without recruiting Neep21 (Figure S6), we examined whether GRASP-1 influences the Neep21/syntaxin 13 complex. Overexpression of GRASP-1 strongly reduced the colocalization between syntaxin 13 and Neep21 (∼15%) compared to control neurons (∼40%) (Figure 11D,G), suggesting that GRASP-1 competes with Neep21 for binding to syntaxin 13, thereby affecting the integrity of the Neep21/syntaxin 13 complex. These data are consistent with the observation that GRASP-1 separates Rab4 from Neep21 endosomal domains.

To evaluate whether syntaxin 13 is important for GRASP-1 association with Rab11 domains, we triple transfected GFP-Rab4, HA-Rab11, and a myc-tagged dominant negative syntaxin 13ΔTM mutant lacking the transmembrane domain. Hippocampal neurons transfected with syntaxin 13ΔTM showed a strong decrease in Rab4/Rab11 colocalization (∼10%) compared to control neurons (∼30%) (Figure 11E,H), while the co-distribution of Rab4 and GRASP-1 was not affected (unpublished data). These data indicate that syntaxin 13 regulates Rab4/GRASP-1 association with Rab11 endosomes.

## Discussion

Complex processes that govern neuronal function have adapted basic cellular pathways to perform the elaborate information processing achieved by the brain. Some of these processes, such as cargo trafficking, require additional layers of control and fine-tuning. Here, we describe a new molecular mechanism for regulating endosomal membrane and receptor recycling by GRASP-1 in neuronal cells. GRASP-1 is a neuronal effector of Rab4, binds syntaxin 13, and couples Rab4 and Rab11 endosomal domains. This mechanism has two distinct roles in neuronal function; first, it is required for AMPAR recycling, and second, it is critical for dendritic spine morphology.

### Regulation of Recycling Endosome Maturation by GRASP-1

**Figure 12:**
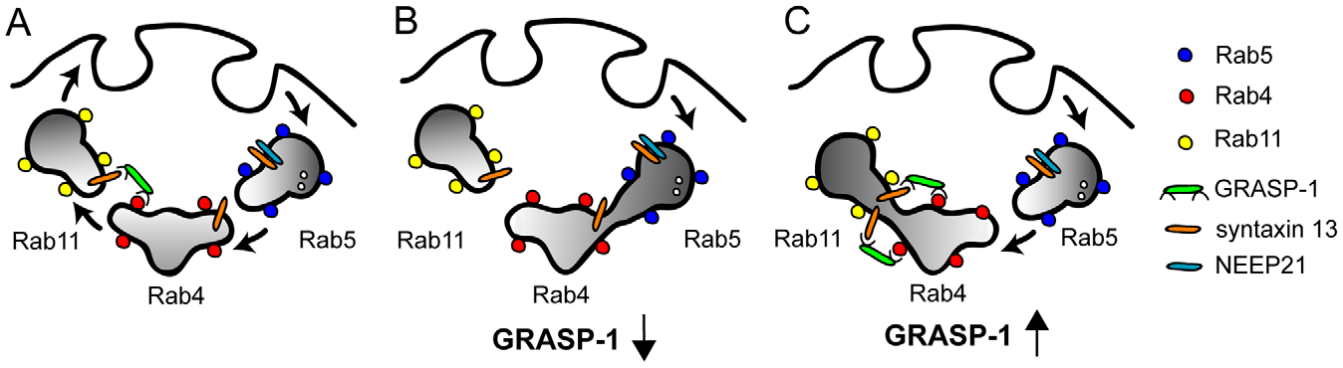
Model for the role of GRASP-1 in endosome recycling. Endosomes can be viewed as mosaic distribution of Rab4, Rab5, and Rab11 domains that dynamically interact via effector proteins and SNAREs. The Rab5 domain allows entry into the early/sorting endosome, whereas the Rab4 and Rab11 domains contain the machinery that is necessary for sorting and recycling membranes and receptors back to the plasma membrane. (A) GRASP-1 binds to Rab4 and syntaxin 13 and couples Rab4 and Rab11 recycling endosomes. The complex formed between GRASP-1 and t-SNARE syntaxin 13 might mediate fusion between Rab4 and Rab11 endosomes. (B) Absence of GRASP-1 interferes with complex formation at the recycling step, causing cargo accumulation in early endosomes, impairment of receptor expression, and changes in spine morphology. (C) Overexpression of GRASP-1 leads to recruitment of syntaxin 13 and strongly couples Rab4 and Rab11 domains, causing accumulation of internalized receptors in recycling endosomes. Consistent with the observed decrease in AMPAR clusters [28], Caspase-3 cleavage of GRASP-1 might separate the N-terminal Rab4 domain from the C-terminal syntaxin 13 binding site and disrupt the coupling between Rab4 and Rab11 domains.

Each organelle carries its own set of Rabs which ensures the specificity of intracellular membrane transport. Ample examples show that Rab GTPases and their effectors can confer directionality to membrane traffic and couple different traffic steps [37]. Here, we show that GRASP-1 is a new component of the molecular machinery that regulates directionality in endosomal trafficking in neurons. First, GRASP-1 is a novel Rab4 effector and binds specifically to its active GTP-bound state. Second, knock-down of GRASP-1 separates Rab4 and Rab11 domains and moves Rab4 in EEA1/Neep21 positive early endosomal structures. Accordingly, knock-down of GRASP-1 mimics the effects of dominant-negative Rab4 and Rab11 on dendritic spine morphology. Third, GRASP-1 overexpression strongly increases Rab4/Rab11 colocalization in both neurons and Hela cells. We propose a model in which GRASP-1 coordinates recycling endosomal maturation (Figure 12). The term *recycling endosome maturation* is used here to discern it from the other endosomal exit routes, such as the degradative multivesicular body/endosome maturation pathway, the retrieval route of mannose 6-phosphate receptors to the trans Golgi network, or the pathway for melanogenic enzymes to melanosomes [38].

How does GRASP-1 couple specific Rab domains? Along the endosomal pathway, bivalent effectors have been found that connect proximal Rab5 and Rab4 domains on early endosomes [25]. Since GRASP-1 binds directly to Rab4 but not to Rab11, additional factors are needed. We found that GRASP-1 binds to endosomal SNARE protein syntaxin 13. Overexpression of GRASP-1 separates syntaxin 13 from Neep21 positive structures and strongly recruits syntaxin 13 to Rab4 positive membranes. Previous studies have shown that syntaxin 13 is involved in recycling of endosomal domains [13],[35] and is enriched in Rab11 endosomal fractions [34]. Syntaxin 13 also has a function together with syntaxin 6 in the fusion of early endosomes in vitro [39],[40]. We found that mutant syntaxin 13 separates Rab4/GRASP-1 and Rab11 positive endosomal domains, suggesting a novel function of syntaxin 13 in the coupling of Rab4 and Rab11 domains by GRASP-1. Since syntaxin 13 is a constituent of the SNARE core complex [35] and involved in membrane fusion [36], it is tempting to speculate that the binding between GRASP-1 and syntaxin 13 recruits the fusion machinery necessary to connect with Rab11 positive membranes. Additional studies are required to determine the precise functional relationship between GRASP-1 binding to syntaxin 13 and the SNARE function of syntaxin 13.

The property to bind Rab4 via the N terminus and syntaxin 13 via the C terminus of GRASP-1 supports the model that membrane bound active Rab4 retains or recruits GRASP-1 on endosomes and forms a complex with syntaxin 13. This sequence of interactions could then structurally and functionally link Rab4 to Rab11 membrane domains (Figure 12). Subsequent recruitment of the other factors on to Rab4-defined membrane domains could strengthen the interaction with Rab11. It has been speculated that the GTPase-activating proteins (GAPs) that act on the upstream Rabs might be effectors of the downstream Rabs [41]. These Rab cascades and conversions might serve as a positive feedback loop to specifically concentrate activated Rab4 on Rab11 positive endosomes. Additional regulation of GRASP-1 by caspase-3 cleavage [28] could separate the N-terminal Rab4 binding domain from the C-terminal syntaxin 13 binding site, potentially disrupting the interaction between Rab4 and Rab11 endosomes (Figure 12).

### Role of GRASP-1 in Endosomal AMPAR Recycling

GRASP-1 was originally found to act as a neuronal Ras GEF and regulate synaptic AMPAR trafficking [28]. We could not measure detectable GEF activity of GRASP-1 for Ras in vivo, by filter binding (unpublished data) or sensitive fluorometric mantGDP assays, nor did we find homology between the GRASP-1 sequence and known rasGEF domains. Here, we provide an alternative model for the role of GRASP-1 in AMPAR traffic and show that GRASP-1 is part of the molecular machinery that controls endosomal membrane receptor recycling in dendrites. Indeed, we show that GRASP-1 colocalizes with internalized AMPARs and that knock-down of GRASP-1 decreases recycling of GluR subunits after AMPA application. Moreover GRASP-1 regulates synaptic plasticity, especially the late phase of LTP in hippocampal slices. Previous results show that Rab11 and syntaxin 13 dominant negative mutants were critical for the entire time course of LTP [12],[22]. We propose that GRASP-1 regulates a particular step in the endosomal trafficking and is important for a specific phase of AMPA receptor recycling (Figure 12). In addition to supplying AMPARs, membrane trafficking from recycling endosomes also mediates the growth of dendritic spines [2],[22],[42]. In accord, GRASP-1 knock-down decreased the total number of protrusions and mushroom-shaped spines and regulates endosomal mobility into dendritic spines. As discussed above, the coupling of endosomal Rab4 and Rab11 domains by GRASP-1 is an attractive possibility to explain the effects on AMPAR recycling and spine morphology.

GRASP-1 binds to the seven PDZ domain-containing scaffolding protein GRIP [28] that transports and stabilizes GluR2 containing AMPAR at synapses and intracellular compartments [5],[43]. Rab4 dominant negative and GRASP-1 knock-down had no effect on GRIP-1 distribution and Rab4/GRASP-1 positive endosomal structures did not recruit endogenous GRIP (unpublished data), suggesting that GRIP functions in an alternative trafficking pathway independent of GRASP-1 or the interaction with GRASP-1 is transient and highly regulated. Interestingly, GRIP also binds to the early endosomal protein Neep21, which is crucial for AMPAR sorting through endosomes [44],[45]. Since neuronal activity determines the phosphorylation status of GRIP and enhances the binding of GRIP and GluR2 with Neep21 [44],[45], it is possible that GRIP is under tight control of specific phosphorylation signaling mechanisms in order to allow for consecutive protein binding and temporal receptor interactions [44],[45]. Additional studies are required to determine the precise role of GRIP in endosomal receptor trafficking.

In contrast to AMPA stimulation, GRASP-1 staining strongly decreased by bath application of NMDA [10],[28]. It has been shown that AMPA and NMDA stimulation induce differential AMPAR sorting; AMPA stimulation allows AMPARs to enter the normal recycling pathway, whereas NMDA stimulation diverts AMPARs to Neep21-positive endosomes and the lysosome degradation pathway [10],[13]. It is tempting to speculate that GRASP-1 in AMPA stimulated neurons allows sorting of internalized AMPARs to the recycling endosomes, while in response to NMDA, absence of GRASP-1 drives receptors to the lysosomes. It is possible that different neuronal stimulatory inputs dynamically control activity of effector complexes and endosomal trafficking pathways. In this model, GRASP-1 might be part of the machinery on endosomes that senses and reacts on NMDA receptor-mediated Ca^2+^ influx, which is of key importance to understanding internalized AMPAR and membrane sorting during plasticity and neuronal circuitry remodeling.

## Materials and Methods

### Antibodies and DNA Constructs

The following primary antibodies were used in this study: rabbit anti-GRASP-1 (JH 2730) [28], rabbit anti-NEEP21 [13], rabbit anti-GRIP1 [43], rabbit anti-Rab4 [46], rabbit anti-GFP [47], rabbit anti-syntaxin 13 [35]. Rabbit anti-Rab11 was generated by immunizing animals with GST-Rab11a and affinity purified on His-Rab11a columns. Anti-GRASP-1 (#5285) was generated by immunizing rabbits with GST-GRASP-1(1–378) and used for immuno electronmicroscopy.

The following antibodies were obtained from commercial sources: rabbit anti-GRASP-1 (AB96361), mouse anti-β-actin, mouse anti-GluR2 (Chemicon), mouse anti-Rab4, mouse anti-EEA1 (BD Biosciences), mouse anti-FLAG, mouse anti-MAP2, mouse anti-αtubulin (Sigma), mouse anti-GFP (Roche), mouse anti-bassoon (Stressgen), rabbit anti-β-galactosidase (MP Biomedicals), mouse anti-β-galactosidase (Promega), rabbit anti-GluR1 (Calbiochem), mouse anti-HA (Roche), rabbit anti-myc (Upstate Biotechnology), mouse LAMP-1 (Stressgen), mouse anti-myc, rabbit anti-Rab5 (Santa Cruz Biotechnology), rabbit anti-syntaxin 13 (Synaptic Systems), human anti-EEA1, and mouse anti-human TfR (ATCC). TfR-594, HRP, and fluorescently labeled secondary antibodies were from Molecular Probes and Jackson Laboratories, and agarose beads conjugated with mouse anti-FLAG antibody were purchased from Sigma. The following mammalian expression plasmids have been described: pRK5-myc-GRASP-1 [28], pEF-Flag-Rab4 and pEF-Flag-Rab5 [48], pEGFP-Rab7 [49], pβactin-HA-β-galactosidase [43], pJPA5-Tfr-GFP [50], Rab3, Rab4, Rab5, Rab7 and Rab11 cDNA in pGEX, pEGFP or pcDNA3 [47],[51]–[54], pGEX-Hras(1–166) and pGEX-cdc25(974–1260) [55], pcDNA3-NEEP21-GFP, pcDNA3-myc-syntaxin 13 [13], pEGFP-Rab11S25N [56], pEGFP-Rab4S22N [57], pGW1-HA-GluR2 [10], pSuper vector [58], and pSuper-GRIP1-shRNA [43]. pMT2HA-rasGRP and pMT2HA-ras were obtained from Hans Bos (Laboratory of Physiological Chemistry, University Medical Center, Utrecht). Syntaxin constructs were obtained as indicated; pGEX-syntaxin1ΔTM and pGEX-syntaxin2ΔTM (Ruud Toonen, CNCR, VU, Amsterdam), pGEX-syntaxin6ΔTM (Suzanne Pfeffer, Stanford School of Medicine), and pGEX-syntaxin13ΔTM (Andrew Peden, CIMR, Cambridge).

GRASP-1 truncation constructs and GRASP-1 mutant lacking aa280–300 were made with PCR from full-length GRASP-1 cDNA 1 [28]. GRASP-1* rescue constructs were prepared by a PCR-based strategy to introduce four silent substitutions in the target site. The target sequence GCTCTCTGAGAAATTGAAA was modified into GCTTTCGGAAAAGTTGAAA. Syntaxin-1A (BC100446; image: 6595634), syntaxin-2 (BC047496; image: 5296500), and syntaxin-6 (BC009944; image: 4122224) cDNA was purchased from Geneservice. For neuronal expression, all cDNAs were subcloned in pGW1- and pβactin-expression vectors with various tags [43]. Myc-syntaxin 13ΔTM (aa1–245) was made by PCR from full-length syntaxin 13 cDNA. The rat GRASP-1 (accession NM_053807) smartpool siRNA (cat# L-096315-01) was from Dharmacon. Another set of three separate siRNAs targeting rat GRASP-1 was purchased from Ambion (cat# AM16798A). GRASP-1-siRNA2 (siRNA ID#192942, GCUCUCUGAGAAAUUGAAAtt) yielded most efficient knock-down in INS1 cells and was cloned in pSuper plasmid for knock-down of GRASP-1 in rat hippocampal neurons. GRASP-1 shRNA#5 (GTCCCAGCACAAAGAAGAA) was designed by using the siRNA selection program at the Whitehead Institute for Biomedical Research [59] (jura.wi.mit.edu/bioc/siRNAext). The sequence for the Luciferase shRNA is CGTACGCGGAATACTTCGA [60].

### Preparation of Tissue Extracts

For tissue Western blots, cerebral cortex, cerebellum, midbrain, spinal cord, kidney, liver, and spleen were dissected from P30 mice. Frozen tissue samples and cultured cells were homogenized in PBS/1%Triton-×100, and then an equal volume of 2× SDS sample buffer was added, and the samples were boiled. Protein concentrations were measured using a BCA protein assay kit (Pierce), and 20 µg of protein was loaded in each lane for a subsequent Western blot analysis.

### GST-Rab Pull-Down Assays

Preparation of pig brain cytosol, purification of GST-Rab fusion proteins, isolation of Rab4GTP-interacting proteins in pull-down assays and binding assays with ^35^S-labeled GRASP-1 were done as described [52],[61]. To determine the Rab4 binding region on GRASP-1, we expressed pRK5-myc GRASP-1 or pGW1-GFP-GRASP-1 truncations in COS-7 cells. Cells were washed in ice-cold PBS and lysed in 20 mM Hepes pH 7.4, 100 mM NaCl, 5 mM MgCl_2_ (lysis buffer) containing 0.5% NP-40, 5 µg/ml leupeptin, 10 µg/ml aprotinin, 1 µg/ml pepstatin, 1 mM PMSF, 20 µM GMP-PNP, and 1 mM DTT. Detergent lysates were shaken for 20 min at 4°C, centrifuged for 10 min at maximum speed in a cooled Eppendorf centrifuge, diluted with lysis buffer to 0.2% NP-40, and incubated with Rab4-GMP-PNP beads for 2 h at 4°C. Beads were washed four times with lysis buffer containing 0.2% NP-40, 20 µM GMP-PNP, and 1 mM DTT. Bound proteins were eluted in Laemmli sample buffer and analyzed by Western blot and detection with anti-myc antibody. To determine whether Rab4, GRASP-1, and syntaxin 13 can form a ternary complex, we transfected COS-7 cells with pGW1-myc-syntaxin 13 with and without pGW1-GFP-GRASP-1. Cells were lysed in 20 mM Na Hepes pH 7.5, 100 mM NaCl, 1% TX-100, and cleared detergent lysates were incubated with GST-Rab4 or GST beads. Beads were washed three times with lysis buffer, and bound protein was assayed by Western blot with monoclonal antibodies against GFP and myc epitope tags.

### Binding Assays with GST-Syntaxins

GST-syntaxin fusion proteins lacking the transmembrane domain were expressed in *Escherichia coli* BL21(DE3), immobilized on GSH beads, and used for binding assays with lysates of COS-7 cells transfected with GFP-GRASP-1(594–837). Binding assay of GST-syntaxin13ΔTM and ^35^S-labeled GRASP-1, produced in a coupled in vitro transcription-translation reaction, was done as described [61]. For mapping the syntaxin 13 binding domain on GRASP-1, we expressed C terminal pGW1-GFP-GRASP-1 constructs in COS-7 cells. The cells were metabolically labeled for 30 min with 0.5 mCi/ml ^35^S-methionine/Pro-Mix (Perkin Elmer), and detergent lysates were then subjected to a GST pull-down assay on GST-syntaxin13ΔTM as described above. Bound proteins were released by boiling the beads 8 min in 0.1 ml 1% SDS/PBS, and GFP-tagged GRASP-1 truncations were immunoprecipitated with a rabbit GFP antibody and analyzed by phosphorimaging as before [62].

### Mass Spectrometry

Eluates were boiled in Laemmli sample buffer, resolved on a 7.5% SDS-PAA gel, and silver-stained. Bands of interest were excised and in-gel digested using modified trypsin (Roche Diagnostics, Indianapolis, IN) in 50 mM ammonium bicarbonate. The peptide mixtures were analyzed by LC/MS/MS using a Q-ToF hybrid mass spectrometer (Micromass, Waters) equipped with a Z-spray source and coupled on-line with a capillary chromatography system. The peptide mixtures were delivered to the system using a Famos autosampler (LC Packing) at 3 µl/min and trapped on an AquaTM C18RP column (Phenomenex; column dimension 1 cm×100 µm i.d., packed in house). The sample was then fractionated onto a C_18_ reverse-phase capillary column (PepMap, LC Packing; column dimension 25 cm×75 µm i.d.) at a flow rate of 150–200 nl/min using a linear gradient of acetonitrile. The mass spectrometer was set up in a data-dependent MS/MS mode where a full scan spectrum (*m*/*z* acquisition range from 400 to 1,600 Da/e) was followed by a tandem mass spectrum (*m*/*z* acquisition range from 100 to 1,800 Da/e). The precursor ions were selected as the most intense peaks of the previous scan. Suitable collision energy was applied depending on the mass and charge of the precursor ion. ProteinLynx software, provided by the manufacturers, was used to analyze raw MS and MS/MS spectra and to generate a peak list which was introduced in the MASCOT MS/MS ion search software for protein identification.

### Immunoprecipitation

COS-7 cells were cotransfected with pEF-FLAG-Rab4 or pEF-FLAG-Rab5 and GRASP-1 constructs and co-immunoprecipitations were done as described [47]. Immune complexes were eluted with FLAG peptide and analyzed by Western blot with a mouse monoclonal antibody against GFP and rabbit antibody against FLAG. For interaction studies between GRASP-1 and syntaxin 13, COS-7 cells were transfected with pGW1-GFP-GRASP-1, and pGW1-myc-syntaxin 1, pGW1-myc-syntaxin 2, or pGW1-myc-syntaxin 13. To determine the region of GRASP-1 that bound to syntaxin13, we transfected COS-7 cells with pGW1-myc-GRASP-1, pGW1-myc-GRASP-1(1–695) or pGW1-myc-GRASP-1(695–837) and pGW1-GFP-syntaxin-13. Cells were lysed in 20 mM Hepes pH 7.4, 200 mM NaCl, 1% NP-40, and protease inhibitors. Detergent lysates were passed 20× through a 27-gauge needle and centrifuged at maximum speed in a cooled Eppendorf centrifuge. The supernatant was incubated for 2 h with Rabbit GFP antibody coated beads at 4°C. Beads were washed four times with 20 mM Hepes pH 7.4, 200 mM NaCl, 1% NP-40, and immune complexes were eluted by heating for 5 min in reducing Laemmli sample buffer. Eluates were resolved by SDS-PAGE and analyzed by Western blot with monoclonal antibody against myc.

### In Vitro GEF Assay

GST-Rab4, H-ras(1–166), GST-GRASP-1(1–594), and GST-cdc25(974–1260) were expressed in *E. coli* CK600K. Bacteria were grown at 37°C until OD_600_ of 0.8. IPTG was added to 1 mM and bacteria were incubated overnight at room temperature. Cells were resuspended in 50 mM Tris HCl pH 7.5, 50 mM NaCl, 5% glycerol, 5 mM DTE, and 5 mM MgCl_2_ and lysed by sonication. Insoluble material was removed by centrifugation at 30,000 g, and in case of GST fusion proteins, the supernatant was loaded on a 20 ml GSH-column (Pharmacia). The column was washed with 5 volumes 50 mM Tris HCl pH 7.5, 400 mM NaCl, 5% glycerol 5 mM MgCl_2_, and 5 mM DTE and 2 volumes of 50 mM Tris HCl pH 7.5, 50 mM NaCl, 2.5% glycerol 10 mM CaCl_2_, 5 mM MgCl_2_, and 5 mM DTE (buffer T). The proteins were cleaved with 80 units of thrombin (Serva) in buffer T on the column and elute with buffer T. Protein containing fractions were concentrated using a Millipore concentrator unit. Further purification was achieved by gel filtration on a Superdex 75 (16/60) column (Pharmacia), equilibrated with 50 mM Tris HCl pH 7.5, 50 mM NaCl, 2.5% glycerol, 5 mM MgCl_2_, and 5 mM DTE. GTPases were loaded with 2′-(3′)-O-(N-methylanthraniloyl)-guanosinediphosphate (mantGDP) as described for rap [55]. Nucleotide exchange reactions were carried out as described [55]. In brief, 200 nM mantGDP loaded GTPase was incubated at 25°C in 50 mM Tris HCl pH 7.5, 50 mM NaCl, 5 mM MgCl_2_, 5 mM DTE, and 5% glycerol in the presence of an 100-fold molar excess of GDP. Exchange factors were added as indicated. The fluorescence intensity was measured over time in a Cary Eclipse Spectrofluorometer (Varian), with excitation at 340 nm and emission at 460 nm.

### In Vivo GEF Assay

COS-7 cells were transfected with pMT2HA-Hras together with pMT2HA-rasGRP, pGW1-myc GRASP-1, or pGW1-myc. Cells expressing HA-ras were incubated with and without 10 ng/ml EGF, and cells co-expressing HA-ras and HA-rasGRP were incubated with or without 10 µM Phorbol 12-Myristate 13-Acetate (PMA). After 5 min, the cells were lysed in 100 mM NaCl, 1% NP40, and 20 mM Hepes pH 7.5. Cleared lysates were incubated for 3 h with GSH beads containing the ras binding domain of Raf-1 [63]. Beads were washed in lysis buffer. Bound HAras-GTP and expression levels of HA-Hras, HA-rasGRP, and myc-GRASP-1 were determined by Western blot with monoclonal HA and myc antibodies, respectively.

### Cultured Cells and Transfection

Hela cells and COS-7 cells were grown in DMEM containing 10% fetal calf serum, antibiotics, and 2 mM glutamine. Transferrin (Tf) uptake experiments in Hela cells were done as described [52]. INS1 cells were grown in RPMI 1640 with the same additions and 0.2 µM Na pyruvate and 50 µM β-mercaptoethanol. Cells were transfected using FuGENE6 (Roche) or Lipofectamine 2000 (Invitrogen) and used in experiments after 16–24 h. siRNAs (100 nM final concentration) were transfected with Lipofectamine 2000 in INS1 cells. After 3 d, cells were lysed and expression level of endogenous GRASP-1 was determined by Western blot.

### Primary Hippocampal Neuron Cultures, Transfection, and Immunohistochemistry

Primary hippocampal cultures were prepared from embryonic day 18 (E18) rat brains [64]. Cells were plated on coverslips coated with poly-L-lysine (30 µg/ml) and laminin (2 µg/ml) at a density of 75,000/well. Hippocampal cultures were grown in Neurobasal medium (NB) supplemented with B27, 0.5 mM glutamine, 12.5 µM glutamate, and penicillin/streptomycin. Hippocampal neurons were transfected using Lipofectamine 2000 (Invitrogen). Briefly, DNA (3.6 µg/well) was mixed with 3 µl Lipofectamine 2000 in 200 µl NB, incubated for 30 min and then added to the neurons in NB at 37°C in 5% CO_2_ for 45 min. Next, neurons were washed with NB and transferred in the original medium at 37°C in 5% CO_2_ for 2–4 d.

For immunohistochemistry, neurons were fixed for 5 min with ice-cold 100% methanol/1mM EGTA at −20°C, followed by 5 min with 4% formaldehyde/4% sucrose in phosphate-buffered saline (PBS) at room temperature. After fixation, cells were washed three times in PBS for 30 min at room temperature and incubated with primary antibodies in GDB buffer (0.2% BSA, 0.8 M NaCl, 0.5% Triton X-100, 30 mM phosphate buffer, pH 7.4) overnight at 4°C. Neurons were then washed three times in PBS for 30 min at room temperature and incubated with Alexa-conjugated secondary antibodies in GDB for 2 h at room temperature and washed three times in PBS for 30 min. Slides were mounted using Vectashield mounting medium (Vector laboratories). Confocal images were acquired using a Zeiss LSM 510 confocal laser-scanning microscope with a 40× or 63× oil objective.

### Surface and Intracellular Staining of AMPA Receptors

Surface staining of endogenous AMPARs was performed as described [10],[43]. Hippocampal neurons were “live” incubated with 10 µg/ml rabbit anti-GluR1 (Calbiochem (1∶8)) and mouse anti-GluR2 (Zymed (1∶80)) N-terminal antibodies at 37°C for 15 min. After brief washing in prewarmed DMEM, neurons were either returned to conditioned medium (for control incubation) or stimulated for 2 min with 100 µM AMPA and 50 µM APV or 50 µM NMDA, washed in DMEM, returned to conditioned medium and incubated for the given time. The neurons were fixed for 5 min with 4% formaldehyde/4% sucrose in PBS, followed by three washes in PBS (30 min at room temperature) and incubated with secondary antibody conjugated to Alexa488 (1∶400) or Alexa568 (1∶400) in GDB buffer without detergent (0.2% BSA, 0.8 M NaCl, 30 mM phosphate buffer, pH 7.4) overnight at 4°C followed by a further three washes in PBS (30 min at room temperature).

The fluorescent-based AMPAR internalization assay was performed as described [10]. Hippocampal neurons transfected with HA-tagged GluR2 subunits were “live” labeled with 10 µg/ml mouse anti-HA antibody (12CA5, Roche) by incubating coverslips in conditioned medium for 10 min at 37°C. After brief washing in prewarmed DMEM, neurons were either returned to conditioned medium (for control incubation) or stimulated for 2 min with 100 µM AMPA and 50 µM APV (selective *NMDA receptor antagonist*) or 50 µM NMDA, returned to conditioned medium and incubated for the given time. Neurons were fixed in 4% formaldehyde/4% sucrose for 8 min at room temperature, and surface-remaining receptors were visualized with Alexa633-conjugated secondary antibody. Internalized receptors were detected with Alexa488-conjugated secondary antibody after permeabilizing cells in methanol (−20°C) for 2 min. To determine colocalization, the neurons were immunostained with antibodies against GRASP-1 in GDB without detergent overnight at 4°C and incubated with Alexa568-conjugated secondary antibodies for 2 h at room temperature.

The AMPAR recycling assay was performed as described [33]. After live staining for surface HA-GluR2 as indicated above, neurons were washed and either returned to conditioned medium (for control incubation) or stimulated for 2 min with 100 µM AMPA and 50 µM APV. The remaining surface anti-HA antibodies were stripped away by stripping buffer (0.5 M NaCl/0.2 M acetic acid) on ice for 4 min and washed extensively with cold TBS (Tris-buffered saline) and returned back to conditioned media at 37°C for 45 min for recycling. After recycling, neurons were fixed in 4% formaldehyde/4% sucrose, and HA-GluR2 recycled back to the surface was detected with Alexa633-conjugated secondary antibodies. Neurons were permeabilized, and internal HA-GluR2 was detected with Alexa568-conjugated secondary antibodies.

### Immunohistochemistry and Confocal Immunofluorescence

The mouse spinal cord was sectioned at 40 µm with a freezing microtome. Sections were processed free floating, employing double-labeling immunofluorescence [65]. The antibodies were diluted in Tris-Buffered-Saline (TBS, pH7.6) containing 1% normal horse serum and 0.2% Triton X-100. Sections stained for immunofluorescence were analyzed with a Zeiss LSM 510 confocal laser-scanning microscope.

### Time-Lapse Live Cell Imaging

During imaging, neurons were maintained at 37°C in standard culture medium in a closed chamber with 5% CO2 (Tokai Hit; INUG2-ZILCS-H2). To visualize mRFP-GRASP-1 and GFP-Rab4 or mRFP-GRASP-1 and GFP-Rab11 in neurons, near-simultaneous dual color (green and red) time-lapse live cell imaging was performed using Total Internal Reflection Fluorescence microscopy (TIRFM) on a Nikon Eclipse TE2000E (Nikon), equipped with Nikon TIRF arm, CFI Apo TIRF 100×1.49 N.A. oil objective (Nikon), Coolsnap camera (Roper Scientific), and controlled by MetaMorph 7.1 software (Molecular Devices). For excitation, the 488 nm laser line of an argon laser (Spectra-Physics Lasers) and a 561 nm laser (Spectra-Physics) were used in combination with a ETGFP/mCherry filter cube (Chroma). A filterwheel (Sutter instruments) with GFP and Cherry emission filters (both Chroma) and synchronized with laser emission alternatingly exposed the camera to GFP or Cherry emission. For glycine treatments, the same microscope was used with regular widefield illumination by a mercury lamp. Glycine treatments were performed as described in [2]. Images of live cells were processed and analyzed using MetaMorph, Adobe Photoshop, or LabVIEW (National Instruments) software.

### Image Analysis and Quantification

Confocal images of transfected neurons were obtained with sequential acquisition settings at the maximal resolution of the microscope (1,024×1,024 pixels). Each image was a z-series of 6–8 images, each averaged 2 times and was chosen to cover the entire region of interested from top to bottom. The resulting z-stack was “flattened” into a single image using maximum projection. Images were not further processed and were of similar high quality to the original single planes. The confocal settings were kept the same for all scans when fluorescence intensity was compared. Morphometric analysis, quantification, and colocalization were performed using MetaMorph software (Universal Imaging Corporation).

#### Morphometric analyses of hippocampal neurons

To visualize the dendritic protrusions, we used β-gal or GFP as an unbiased cell-fill. Because protrusions often crossed several z planes, we took series of stacks from the bottom to the top of all dendrites and used the LSM software to generate image projections for quantitative analyses. All morphological experiments were repeated at least three times with an *n*>7 for individual experiments and were analyzed in a double-blind manner. Between 150 and 300 protrusions were scored for every neuron and expressed per 10 µm length of dendrite. Measurements of length and width of the protrusions were performed as described previously [66] and were classified based on the ratio of spine head width to protrusion length according to the following ratios: the spine whose width was equal to or more than half the size of its length was judged as standard mushroom spine. The protrusion whose width was smaller than half the size of its length was judged as filopodia or thin spine. In those cases where the total length of the spine could not be adequately seen or its length was >5 µm, protrusions were excluded from analysis.

#### Quantification of TfR-GFP distribution in spines

Measurements of TfR-GFP localization in spines and dendrites was performed as described [2]. Confocal images of hippocampal neurons filled with β-gal (red) and labeled for TfR-GFP were analyzed using Metamorph software. The dendritic localization of TfR positive structures relative to spines was categorized according to the presence of GFP signal at the base (a), in the neck (b), or in the head (c) of spines.

#### Colocalization of fluorescent signals in dendrites and cell body

Colocalization of two fluorescent signals was determined using “colocalization” module in Metamorph software as described [10]. The colocalization module provides intensity measurements of the region overlap between signals in red and green channels of image projections. To minimize random overlap due to projection of confocal images, a single optical section from the z series stack that showed the largest amount of fluorescent signals was used to determine the degree of colocalization in the cell body. Statistical analysis was performed with Student's *t* test assuming two-tailed distribution and unequal variation. *n* was defined as the number of transfected cells.

#### Quantification of surface and internalized AMPARs

To measure AMPAR internalization and recycling, images for all conditions in individual experiments were analyzed by using identical microscope settings. Images from each experiment were thresholded, and total staining intensity of surface and internalized GluR1 and/or GluR2 was measured along selected dendritic areas using Metamorph. The internalization index refers to intracellular fluorescence divided by surface fluorescence normalized to untreated control neurons. For experiments comparing internalized and surface staining of AMPARs, dendritic areas were manually drawn and staining intensity in the same selected area was measured in the different channels. The recycling index was calculated as the ratio of surface fluorescence divided by the internalized fluorescence and normalized to unstimulated wild-type control neurons.

#### Analysis of morphological changes upon glycine treatment

For each protrusion, length and maximum width were measured in MetaMorph. Only protrusions longer than 7 pixels (450 nm) were included in the analysis. Protrusions were identified as spines if their width was greater than half their length or greater than 10 pixels (645 nm). Spine growth was probed as the change in sum of spine widths per 10 um and comprises both addition of new spines and growth of pre-existing spines.

### Immuno Electronmicroscopy

Hippocampal neurons (DIV>21) were fixed in paraformaldehyde or a mixture of paraformaldehyde/glutaraldehyde and processed for immunoelectronmicroscopy on ultrathin cryosections as described [67]. Sections were labeled with mouse monoclonal antibody against rab4, and rabbit antibodies against syntaxin 13 [35], or GRASP-1 (#5285).

### Electrophysiology

Electrophysiological recordings were carried out from organotypic slice cultures as described [68]. Neurons were transfected using a biolistic gene gun (Bio Rad) at DIV 3–4 (100 µg DNA; 90% of the construct to test; 10% pCAG-EGFP). Electrophysiological recordings were performed at 5–6 d after transfection. Recordings were carried out in solution containing NaCl, 119 (mM); KCl, 2.5; CaCl_2_, 4; MgCl_2_, 4; NaHCO_3_, 26; NaH_2_PO4, 1; glucose, 11; picrotoxin, 0.15; and 2-chloroadenosine, 0.01 for measuring AMPAR- and NMDAR-EPSC and 0.002 for LTP experiments with 5% CO_2_/95% O_2_, at pH7.4. Whole-cell recordings were made simultaneously from a pair of CA1 pyramidal neurons, one transfected and one untransfected, and synaptic responses were evoked by tungsten bipolar electrode placed in CA1 stratum radiatum area with the frequency of 0.2 Hz. AMPAR-mediated EPSCs were measured at −60 mV and NMDAR-EPSCs were recorded at −40 mV in the presence of NBQX (0.01 mM). AMPAR-EPSCs for LTP experiment were measured at −80 mV and LTP was induced by pairing 2 Hz stimulation with depolarization of the postsynaptic cell to 0 mV for 100 s. Statistical significance was evaluated with the Mann-Whitney test (Figure 5A,B) and *t* test (Figure 5C,D).

## Supporting Information

Figure S1 **Localization of Rab4 and GRASP in vivo.** Mouse spinal cord sections of 40 µm were double-labeled for endogenous GRASP-1 (green) and Rab4 (red). Sections were examined on a Zeiss LSM510 at low magnification to obtain the overview image (top row) or high magnification (bottom row). Arrowheads denote colocalization between GRASP-1 and Rab4 as also shown in the inset with merged colors.
(3.04 MB TIF)

Figure S2 **Rab4 dominant negative mutant affects GRASP-1 localization.** (A–C) Representative images of hippocampal neurons transfected with GDP-bound dominant negative mutant GFP-Rab4S22N at DIV13 for 2 d and stained for endogenous GRASP-1 (A), syntaxin 13 (B), or Neep21 (C). Note that in the neurons transfected with Rab4S22N, almost no GRASP-1 puncta are present in somatodendritic compartments while the localization of syntaxin 13 and Neep21 is unchanged. Arrows indicate transfected neurons in the red channel. Bar is 10 µm. (D–F) Quantification of GRASP-1 fluorescence intensities in cell body of hippocampal neurons transfected as indicated in (A–C). Graphs show mean ± SEM normalized to neighboring neurons. *** *p*<0.0005.
(3.54 MB TIF)

Figure S3 **GRASP-1 shRNA suppresses expression of GRASP-1.** (A) Representative images of hippocampal neurons cotransfected at DIV13 with GFP and either pSuper, pSuper-GRASP-1-shRNA#2, or -shRNA#5 and visualized after 4 d with rabbit antibody against GRASP-1 (red) and GFP (green). Cell body (inset) is enlarged to show loss of GRASP-1 immunoreactivity in GRASP-1-shRNA transfected neurons. Bar is 10 µm. (B) Quantification of GRASP-1 fluorescence intensities in cell body and dendrites of hippocampal neurons transfected at DIV13 for 4 d with GFP and either pSuper, pSuper-GRASP-1-shRNA#2, or -shRNA#5. Staining was done with two distinct rabbit anti-GRASP-1 antibodies: clone JH 2730 and AB96361. Graph shows mean ± SEM normalized to pSuper control neurons. *** *p*<0.0005. (C) Western blot of lysates prepared from INS-1 cells transfected with 100 nM (final concentration) of three siRNAs (Ambion), a smartpool (Dharmacon), or control scrambled siRNA (Dharmacon) for 3 d. siRNA#2 and the smartpool reduced GRASP-1 expression to 15% and 23%, respectively. We cloned the siRNA#2 sequence in pSuper in order to generate pSuper-GRASP-1-shRNA#2 (A,B).
(3.01 MB TIF)

Figure S4 **Internalized HA-GluR2 colocalizes with GRASP-1.** (A) Representative merge image of surface HA-GluR2 (blue) and internalized HA-GluR2 (green) in soma and dendrites of hippocampal neurons labeled for GRASP-1 (red) after 0, 10, and 30 min 100 µM AMPA plus 50 µM APV (AMPA) stimulation. (B) Quantification of the percentage of colocalization of internalized GluR2 with GRASP1 after AMPA/APV treatment at different time points. Each data point represents mean±S.E.M. (5 neurons for each time point). (C) Representative images of neurons triple transfected at DIV13 with GFP and HA-GluR2 and either pSuper control vector or pSuper-GRASP-1-shRNA#2. After 4 d, neurons are “live” labeled with anti-HA antibody for 15 min, followed by 10 min incubation in conditioned medium (control, no treatment) or 2 min incubation in conditioned medium containing 100 µM AMPA plus 50 µM APV (AMPA) followed by additional 8 min in conditioned medium. The neurons are stained for surface and internalized HA-GluR2. (D) Quantification of intracellular accumulation assays, measured as the ratio of internalized/surface fluorescence (internalization index), normalized to GluR2 10 min control (no treatment). Graph shows mean ± S.E.M. (10 neurons for each condition). (E) Representative merge images of neurons cotransfected at DIV13 with HA-GluR2 and either pSuper control vector or pSuper-GRASP-1-shRNA#2 and stained for internalized HA-GluR2 (red) and lysosomal marker Lamp1 (green) in the cell body after stimulation for 30 min with AMPA. (F) Quantification of the percentage of colocalization of internalized GluR2 with Lamp1 as indicated in (E). Graph shows mean ± S.E.M. (5 neurons each). ** *p*<0.005.
(2.15 MB TIF)

Figure S5 **GRASP-1 colocalizes with Rab4 and Rab11 in Hela cells.** (A) Hela cells co-transfected with myc-GRASP-1 and GFP-Rab4, GFP-Rab5, GFP-Rab7, or GFP-Rab11. Bar is 10 µm. (B) Percentage of colocalization between GRASP-1 and Rab proteins in Hela cells as indicated in (A).
(2.99 MB TIF)

Figure S6 **GRASP-1 colocalizes with endogenous recycling endosome markers.** Representative images of cell bodies of hippocampal neurons transfected with GFP-GRASP-1 and labeled with anti-Rab4, anti-Rab5, anti-Rab11, anti-NEEP21, or anti-syntaxin 13 antibodies (all red).
(2.27 MB DOC)

Figure S7 **GRASP-1 regulates EEA1 distribution in Hela cells.** (A) Percentage of colocalization between EEA1 and Rab4 or Rab5 in Hela cells with and without transfected myc-GRASP-1 as shown in (C,D). Error bars indicate S.E.M. *** *p*<0.0005. (B) Hela cells transfected with myc-GRASP-1 and double labeled with anti-EEA1 (red) and anti-myc (green) antibodies. Note the lack of colocalization between EEA1 and GRASP1. Bar is 10 µm. (C–D) Hela cells co-transfected with GFP-Rab4 (C) or GFP-Rab5 (D) with and without myc-GRASP-1. Cells were labeled with anti-EEA1 (red) and anti-myc (blue) antibodies. Bar is 10 µm.
(2.50 MB TIF)

Figure S8 **GRASP-1 segregates Rab4 from NEEP21 positive endosomal membranes.** (A) Representative images of hippocampal neurons double labeled with anti-EEA1 (green) and anti-NEEP21 (red) antibodies. Dendritic segments are enlarged to show the distribution of the markers (bottom). (B,C) Representative images of dendrites of hippocampal neurons cotransfected at DIV13 for 4 d with GFP-Rab5 (B) or GFP-TfR (C) and labeled with anti-NEEP21 (red). (D) Representative images of dendrites of hippocampal neurons cotransfected at DIV13 for 4 d with GFP-Rab4 and pSuper control vector, myc-GRASP-1, or pSuper-GRASP-1-shRNA#2 and labeled with anti-NEEP21 (red) and anti-myc (blue) antibodies.
(1.30 MB TIF)

Figure S9 **GRASP-1 coincides with Rab4 and syntaxin 13 in Hela cells.** (A) Hela cells co-transfected with GFP-GRASP-1 and myc-syntaxin 1, myc-syntaxin 2, or myc-syntaxin 13. (B) Hela cells triple transfected with GFP-GRASP-1, myc-syntaxin 13, and HA-Rab4. Bar is 10 µm.
(3.70 MB DOC)

Figure S10 **Both N and C terminus are necessary for GRASP-1 localization to endosomes.** Hela cells transfected with full-length mRFP-GRASP-1 (1–837) or truncated mRFP-GRASP-1 constructs and labeled with anti-TfR antibodies (green). Bar is 10 µm.
(5.64 MB TIF)

Video S1 **GFP-Rab4 (left) and mRFP-GRASP-1 (right) in hippocampal neurons visualized using TIRF.** This video corresponds to Figure 2G. Total time 3 min. Acquired at 0.5 frame per second. 30× sped up.
(3.88 MB AVI)

Video S2 **GFP-Rab4 (left) and mRFP-GRASP-1 (middle) in hippocampal neurons visualized using TIRF.** This video corresponds to Figure 2G. Right movie shows GFP and RFP in green and red, respectively. Total time 3 min. Acquired at 0.5 frame per second. 30× sped up.
(1.70 MB AVI)

Video S3 **GFP-Rab11 (left) and mRFP-GRASP-1 (right) in hippocampal neurons visualized using TIRF.** This video corresponds to Figure 7B. Total time 3:22 (min:s). Acquired at 1 frame per second. 30× sped up.
(5.52 MB AVI)

Video S4 **GFP-Rab11 (left) and mRFP-GRASP1 (middle) in hippocampal neurons visualized using TIRF.** This video corresponds to Figure 7B. Right movie shows GFP and RFP in green and red, respectively. Total time 3:22 (min:s). Acquired at 1 frame per second. 30× sped up.
(1.01 MB AVI)

## Abbreviations

AMPA: alpha-amino-3-hydroxy-5-methyl-4-isoxazolepropionic acid
AMPAR: alpha-amino-3-hydroxy-5-methyl-4-isoxazolepropionic acid receptor
APV: 2-amino-5-phosphonopentanoic acid
β-gal: β-galactosidase
DIV: days in vitro
EPSC: excitatory postsynaptic current
GAP: GTPase-activating protein
GEF: guanine nucleotide exchange factor
GFP-TfR: GFP-tagged transferrin receptor
GluR: glutamate receptor
GRASP-1: GRIP-associated protein-1
GRIP: glutamate receptor interacting protein
GRP: guanine nucleotide releasing protein
GST: glutathione S-transferase
JNK: Jun-N-terminal kinase
LTD: long-term depression
LTP: long-term potentiation
NB: neurobasal
NEEP21: neuronal endosome enriched protein of 21 kDa
NMDAR: n-methyl-D-aspartic acid receptor
PBS: phosphate-buffered saline
PMA: phorbol myristate acetate
PSD: post synaptic density
shRNA: short hairpin RNA
TBS: Tris-Buffered Saline
TIRFM: Total Internal Reflection Fluorescence microscopy
