# Correction: Neuron Specific Rab4 Effector GRASP-1 Coordinates Membrane Specialization and Maturation of Recycling Endosomes

**DOI:** 10.1371/annotation/b17dfb99-8809-4c5a-86c2-c2a0f7ca7f5e

**Published:** 2010-09-02

**Authors:** Casper C. Hoogenraad, Ioana Popa, Kensuke Futai, Emma Martinez-Sanchez, Phebe S. Wulf, Thijs van Vlijmen, Bjorn R. Dortland, Viola Oorschot, Roland Govers, Maria Monti, Albert J. R. Heck, Morgan Sheng, Judith Klumperman, Holger Rehmann, Dick Jaarsma, Lukas C. Kapitein, Peter van der Sluijs

The fourth author's name was spelled incorrectly. The correct name is: Emma Martinez-Sanchez. The correct citation is: Hoogenraad CC, Popa I, Futai K, Martinez-Sanchez E, Wulf PS, et al. (2010) Neuron Specific Rab4 Effector GRASP-1 Coordinates Membrane Specialization and Maturation of Recycling Endosomes. PLoS Biol 8(1): e1000283. doi:10.1371/journal.pbio.1000283

