# Supplementary figures and images for: Neuron Specific Rab4 Effector GRASP-1 Coordinates Membrane Specialization and Maturation of Recycling Endosomes
Source: PLoS Biol. 2010 Jan 19;8(1):e1000283. doi: 10.1371/journal.pbio.1000283 (PMC2808209; doi:10.1371/journal.pbio.1000283)

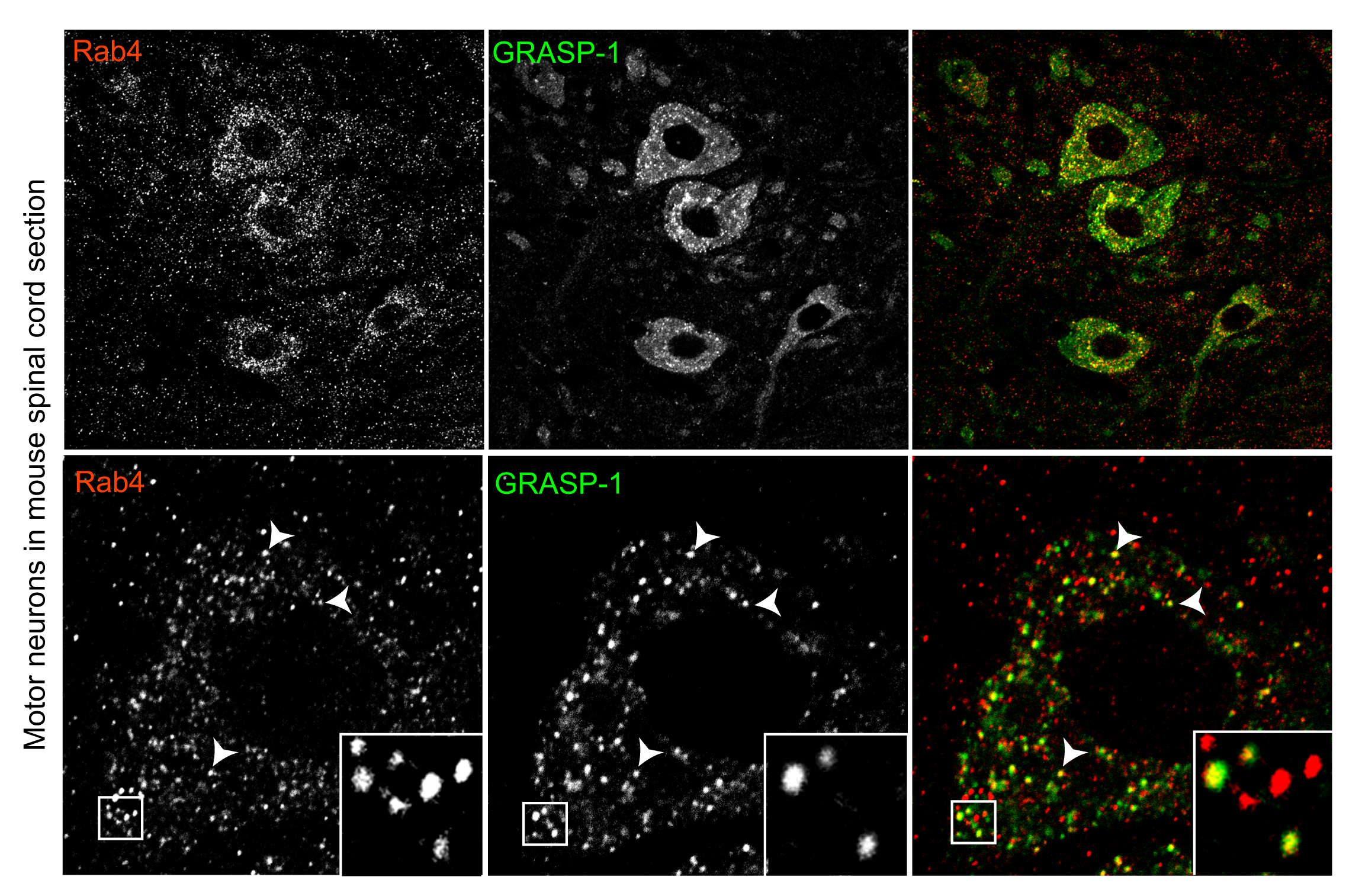

Supplement: Figure S1 — Localization of Rab4 and GRASP in vivo. Mouse spinal cord sections of 40 µm were double-labeled for endogenous GRASP-1 (green) and Rab4 (red). Sections were examined on a Zeiss LSM510 at low magnification to obtain the overview image (top row) or high magnification (bottom row). Arrowheads denote colocalization between GRASP-1 and Rab4 as also shown in the inset with merged colors. (3.04 MB TIF) [file pbio.1000283.s001.tif]

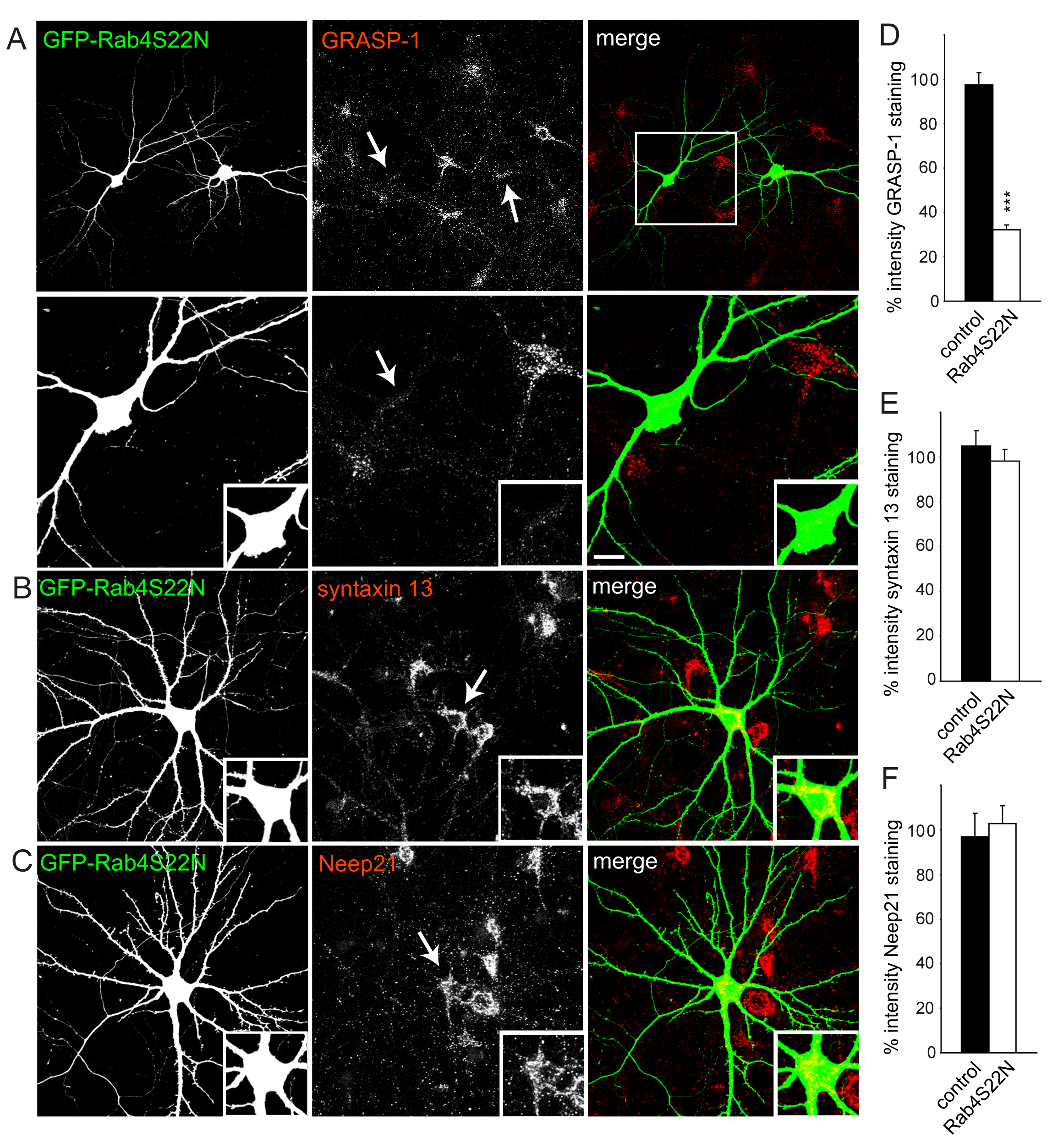

Supplement: Figure S2 — Rab4 dominant negative mutant affects GRASP-1 localization. (A–C) Representative images of hippocampal neurons transfected with GDP-bound dominant negative mutant GFP-Rab4S22N at DIV13 for 2 d and stained for endogenous GRASP-1 (A), syntaxin 13 (B), or Neep21 (C). Note that in the neurons transfected with Rab4S22N, almost no GRASP-1 puncta are present in somatodendritic compartments while the localization of syntaxin 13 and Neep21 is unchanged. Arrows indicate transfected neurons in the red channel. Bar is 10 µm. (D–F) Quantification of GRASP-1 fluorescence intensities in cell body of hippocampal neurons transfected as indicated in (A–C). Graphs show mean ± SEM normalized to neighboring neurons. *** p<0.0005. (3.54 MB TIF) [file pbio.1000283.s002.tif]

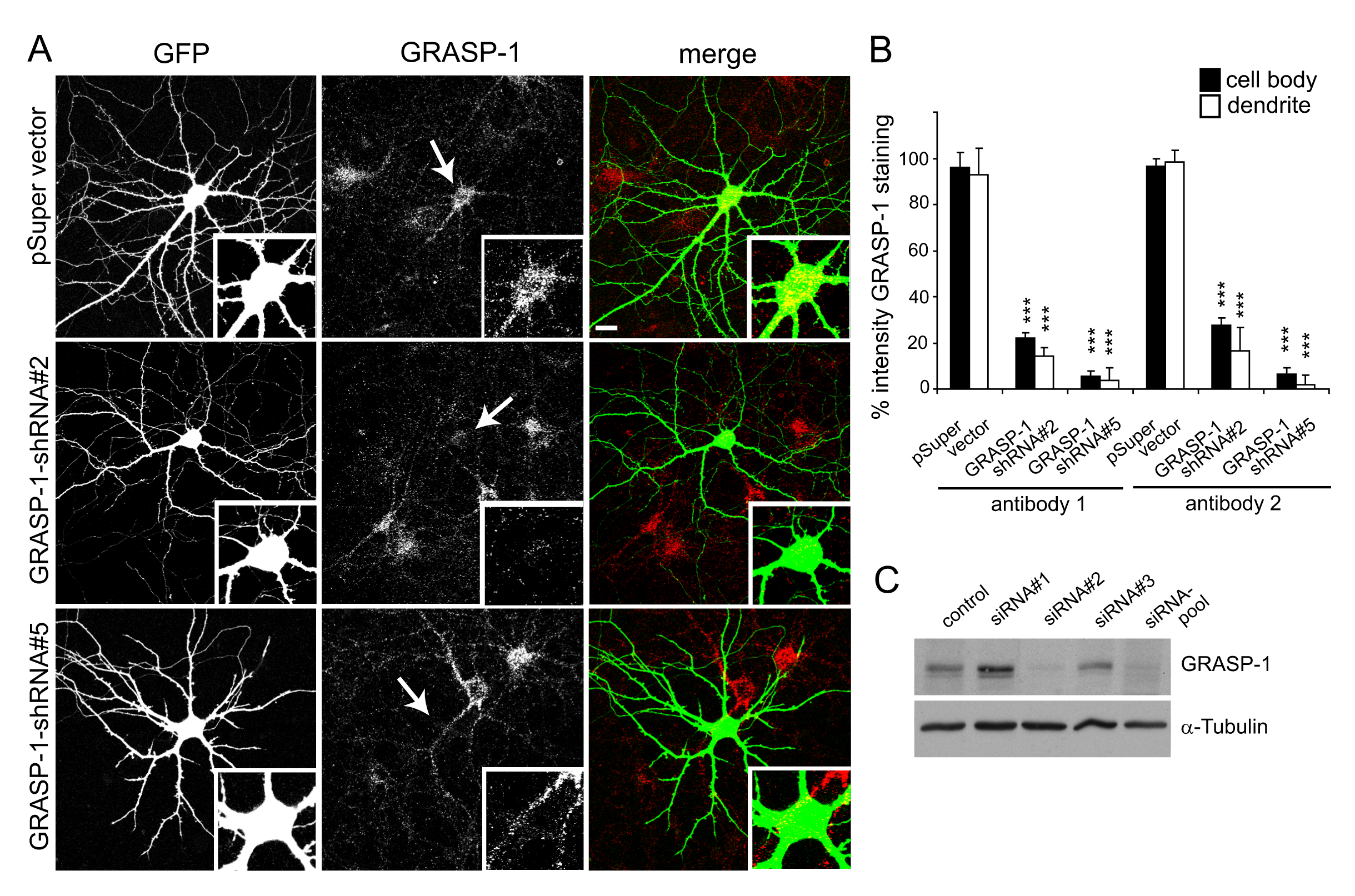

Supplement: Figure S3 — GRASP-1 shRNA suppresses expression of GRASP-1. (A) Representative images of hippocampal neurons cotransfected at DIV13 with GFP and either pSuper, pSuper-GRASP-1-shRNA#2, or -shRNA#5 and visualized after 4 d with rabbit antibody against GRASP-1 (red) and GFP (green). Cell body (inset) is enlarged to show loss of GRASP-1 immunoreactivity in GRASP-1-shRNA transfected neurons. Bar is 10 µm. (B) Quantification of GRASP-1 fluorescence intensities in cell body and dendrites of hippocampal neurons transfected at DIV13 for 4 d with GFP and either pSuper, pSuper-GRASP-1-shRNA#2, or -shRNA#5. Staining was done with two distinct rabbit anti-GRASP-1 antibodies: clone JH 2730 and AB96361. Graph shows mean ± SEM normalized to pSuper control neurons. *** p<0.0005. (C) Western blot of lysates prepared from INS-1 cells transfected with 100 nM (final concentration) of three siRNAs (Ambion), a smartpool (Dharmacon), or control scrambled siRNA (Dharmacon) for 3 d. siRNA#2 and the smartpool reduced GRASP-1 expression to 15% and 23%, respectively. We cloned the siRNA#2 sequence in pSuper in order to generate pSuper-GRASP-1-shRNA#2 (A,B). (3.01 MB TIF) [file pbio.1000283.s003.tif]

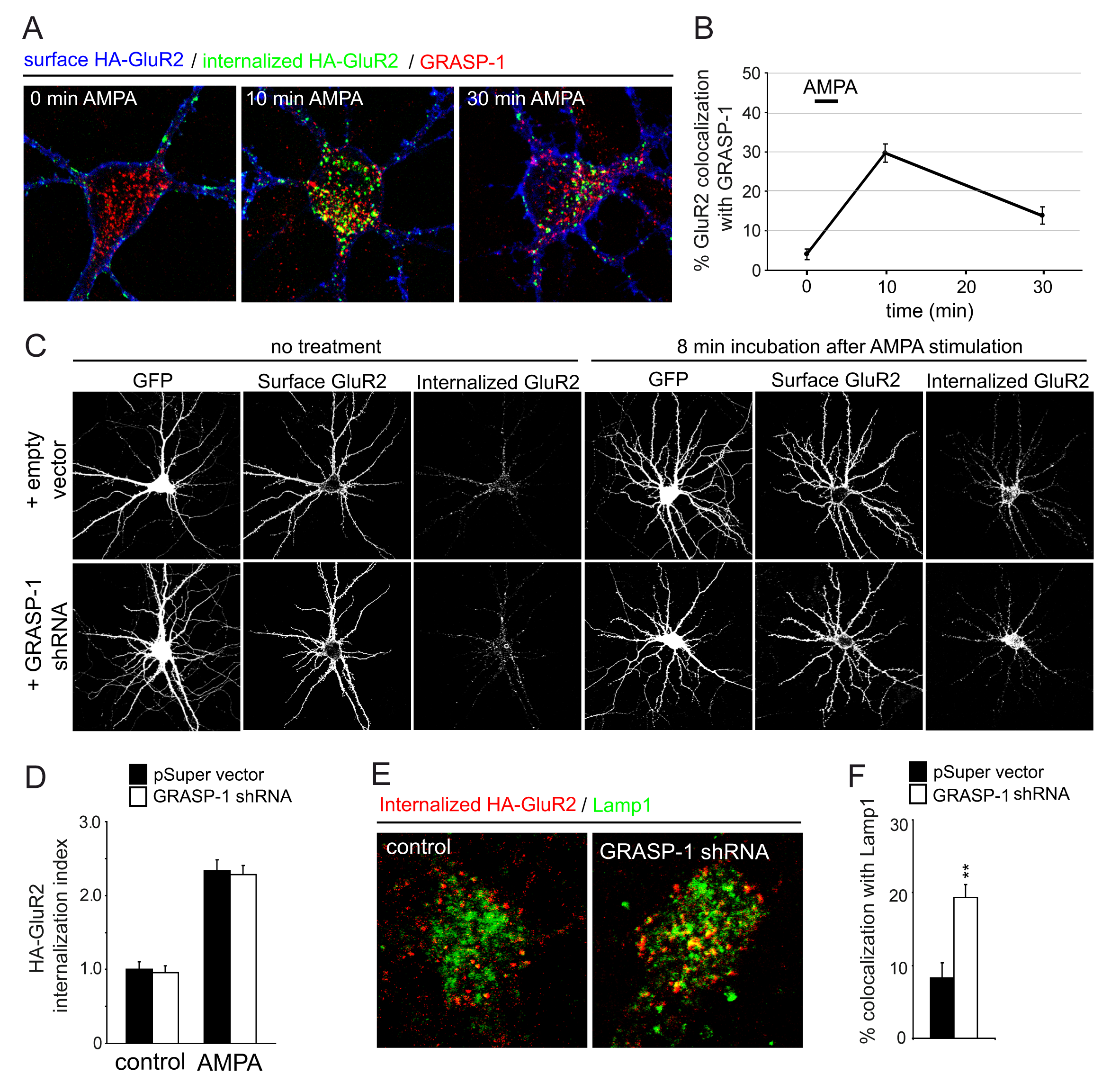

Supplement: Figure S4 — Internalized HA-GluR2 colocalizes with GRASP-1. (A) Representative merge image of surface HA-GluR2 (blue) and internalized HA-GluR2 (green) in soma and dendrites of hippocampal neurons labeled for GRASP-1 (red) after 0, 10, and 30 min 100 µM AMPA plus 50 µM APV (AMPA) stimulation. (B) Quantification of the percentage of colocalization of internalized GluR2 with GRASP1 after AMPA/APV treatment at different time points. Each data point represents mean±S.E.M. (5 neurons for each time point). (C) Representative images of neurons triple transfected at DIV13 with GFP and HA-GluR2 and either pSuper control vector or pSuper-GRASP-1-shRNA#2. After 4 d, neurons are “live” labeled with anti-HA antibody for 15 min, followed by 10 min incubation in conditioned medium (control, no treatment) or 2 min incubation in conditioned medium containing 100 µM AMPA plus 50 µM APV (AMPA) followed by additional 8 min in conditioned medium. The neurons are stained for surface and internalized HA-GluR2. (D) Quantification of intracellular accumulation assays, measured as the ratio of internalized/surface fluorescence (internalization index), normalized to GluR2 10 min control (no treatment). Graph shows mean ± S.E.M. (10 neurons for each condition). (E) Representative merge images of neurons cotransfected at DIV13 with HA-GluR2 and either pSuper control vector or pSuper-GRASP-1-shRNA#2 and stained for internalized HA-GluR2 (red) and lysosomal marker Lamp1 (green) in the cell body after stimulation for 30 min with AMPA. (F) Quantification of the percentage of colocalization of internalized GluR2 with Lamp1 as indicated in (E). Graph shows mean ± S.E.M. (5 neurons each). ** p<0.005. (2.15 MB TIF) [file pbio.1000283.s004.tif]

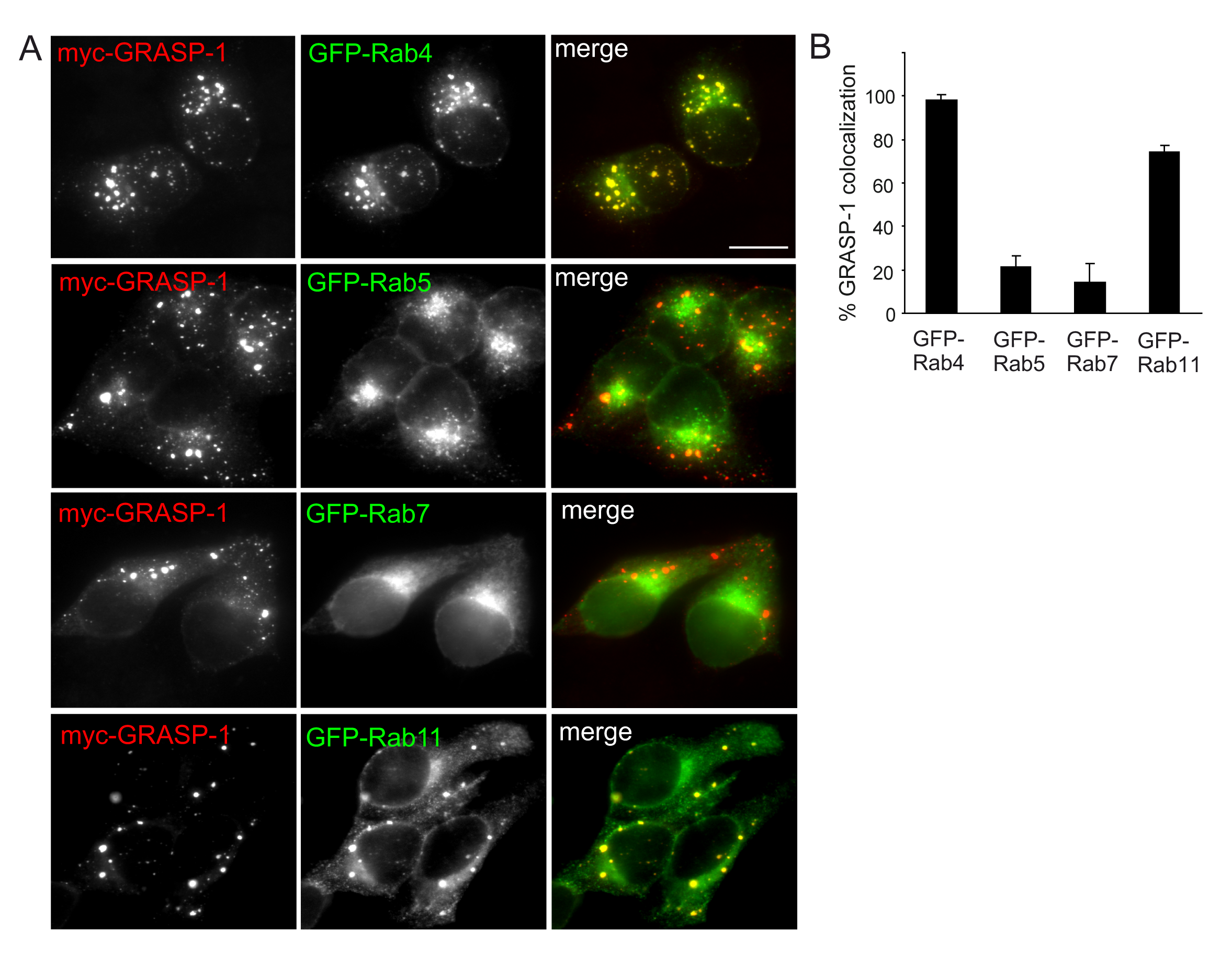

Supplement: Figure S5 — GRASP-1 colocalizes with Rab4 and Rab11 in Hela cells. (A) Hela cells co-transfected with myc-GRASP-1 and GFP-Rab4, GFP-Rab5, GFP-Rab7, or GFP-Rab11. Bar is 10 µm. (B) Percentage of colocalization between GRASP-1 and Rab proteins in Hela cells as indicated in (A). (2.99 MB TIF) [file pbio.1000283.s005.tif]

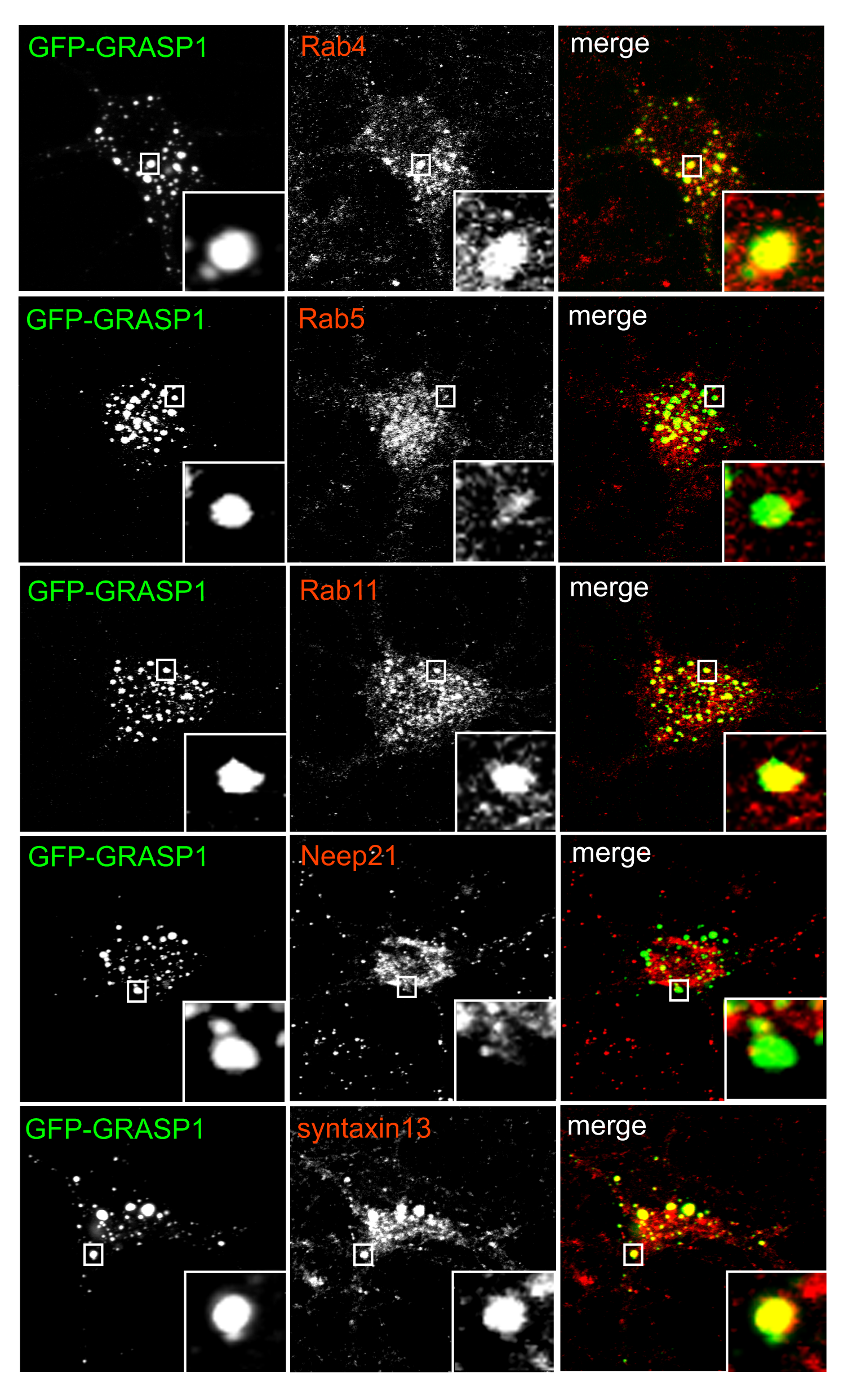

Supplement: Figure S6 — GRASP-1 colocalizes with endogenous recycling endosome markers. Representative images of cell bodies of hippocampal neurons transfected with GFP-GRASP-1 and labeled with anti-Rab4, anti-Rab5, anti-Rab11, anti-NEEP21, or anti-syntaxin 13 antibodies (all red). (2.27 MB DOC) [file pbio.1000283.s006.tif]

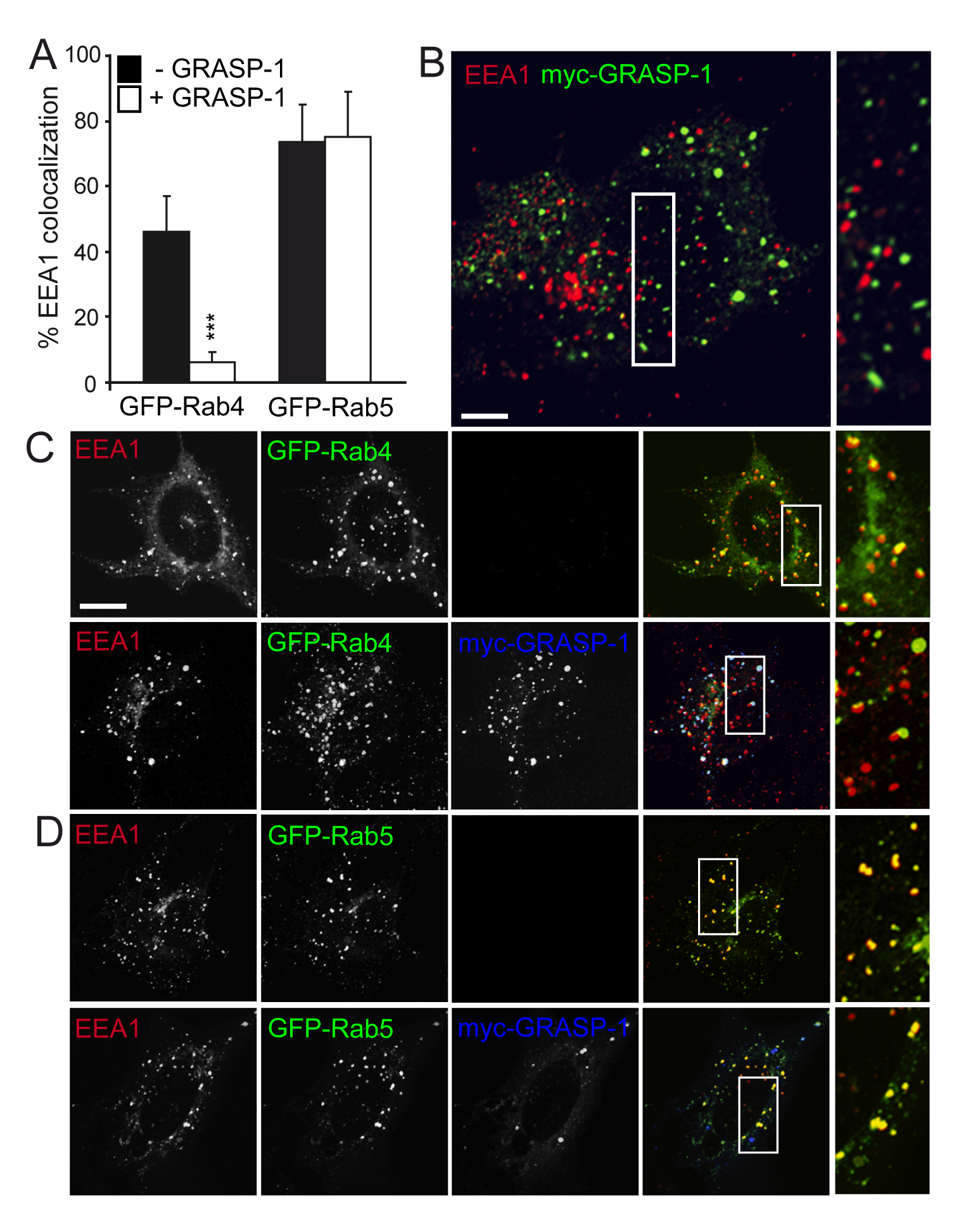

Supplement: Figure S7 — GRASP-1 regulates EEA1 distribution in Hela cells. (A) Percentage of colocalization between EEA1 and Rab4 or Rab5 in Hela cells with and without transfected myc-GRASP-1 as shown in (C,D). Error bars indicate S.E.M. *** p<0.0005. (B) Hela cells transfected with myc-GRASP-1 and double labeled with anti-EEA1 (red) and anti-myc (green) antibodies. Note the lack of colocalization between EEA1 and GRASP1. Bar is 10 µm. (C–D) Hela cells co-transfected with GFP-Rab4 (C) or GFP-Rab5 (D) with and without myc-GRASP-1. Cells were labeled with anti-EEA1 (red) and anti-myc (blue) antibodies. Bar is 10 µm. (2.50 MB TIF) [file pbio.1000283.s007.tif]

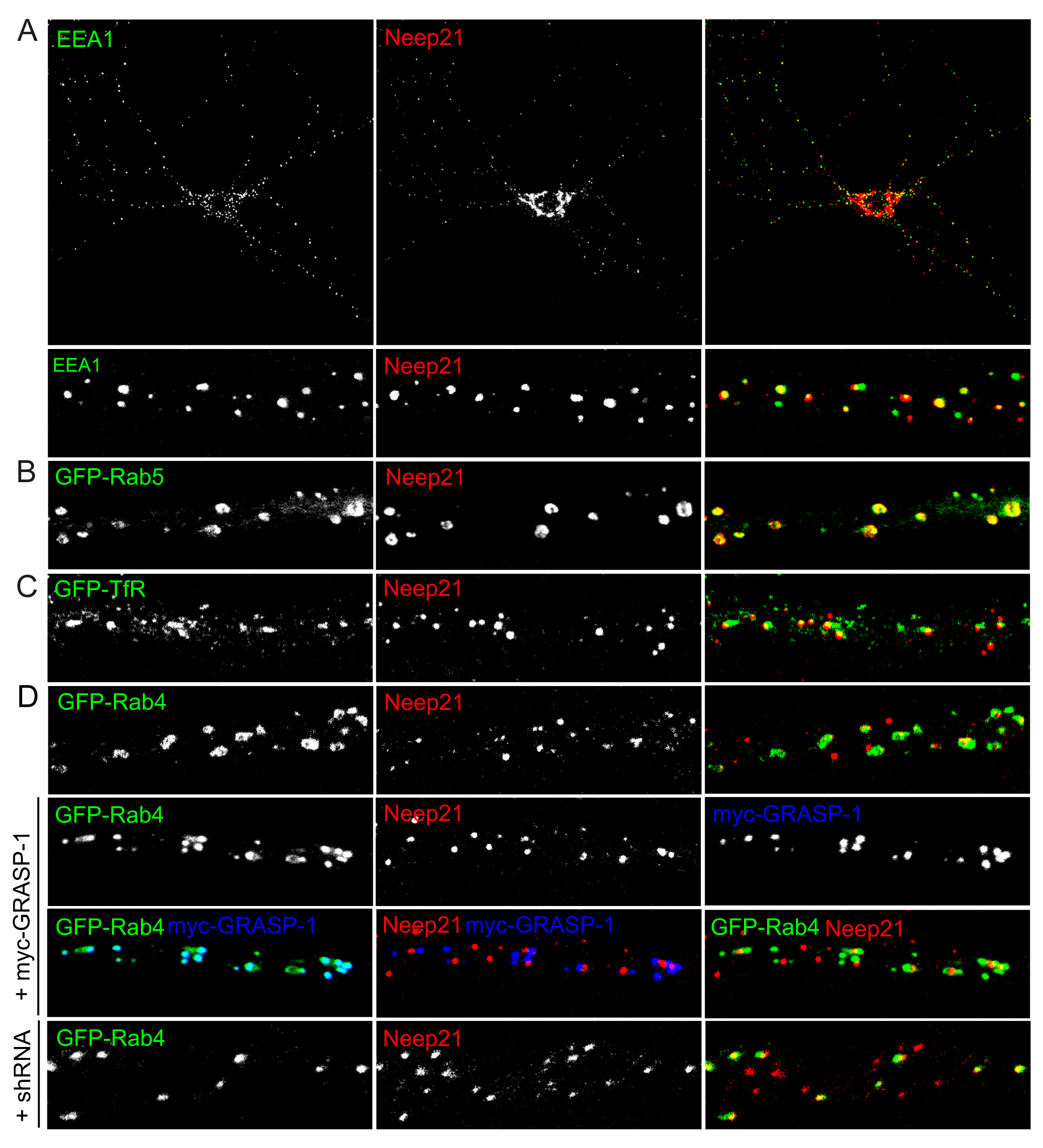

Supplement: Figure S8 — GRASP-1 segregates Rab4 from NEEP21 positive endosomal membranes. (A) Representative images of hippocampal neurons double labeled with anti-EEA1 (green) and anti-NEEP21 (red) antibodies. Dendritic segments are enlarged to show the distribution of the markers (bottom). (B,C) Representative images of dendrites of hippocampal neurons cotransfected at DIV13 for 4 d with GFP-Rab5 (B) or GFP-TfR (C) and labeled with anti-NEEP21 (red). (D) Representative images of dendrites of hippocampal neurons cotransfected at DIV13 for 4 d with GFP-Rab4 and pSuper control vector, myc-GRASP-1, or pSuper-GRASP-1-shRNA#2 and labeled with anti-NEEP21 (red) and anti-myc (blue) antibodies. (1.30 MB TIF) [file pbio.1000283.s008.tif]

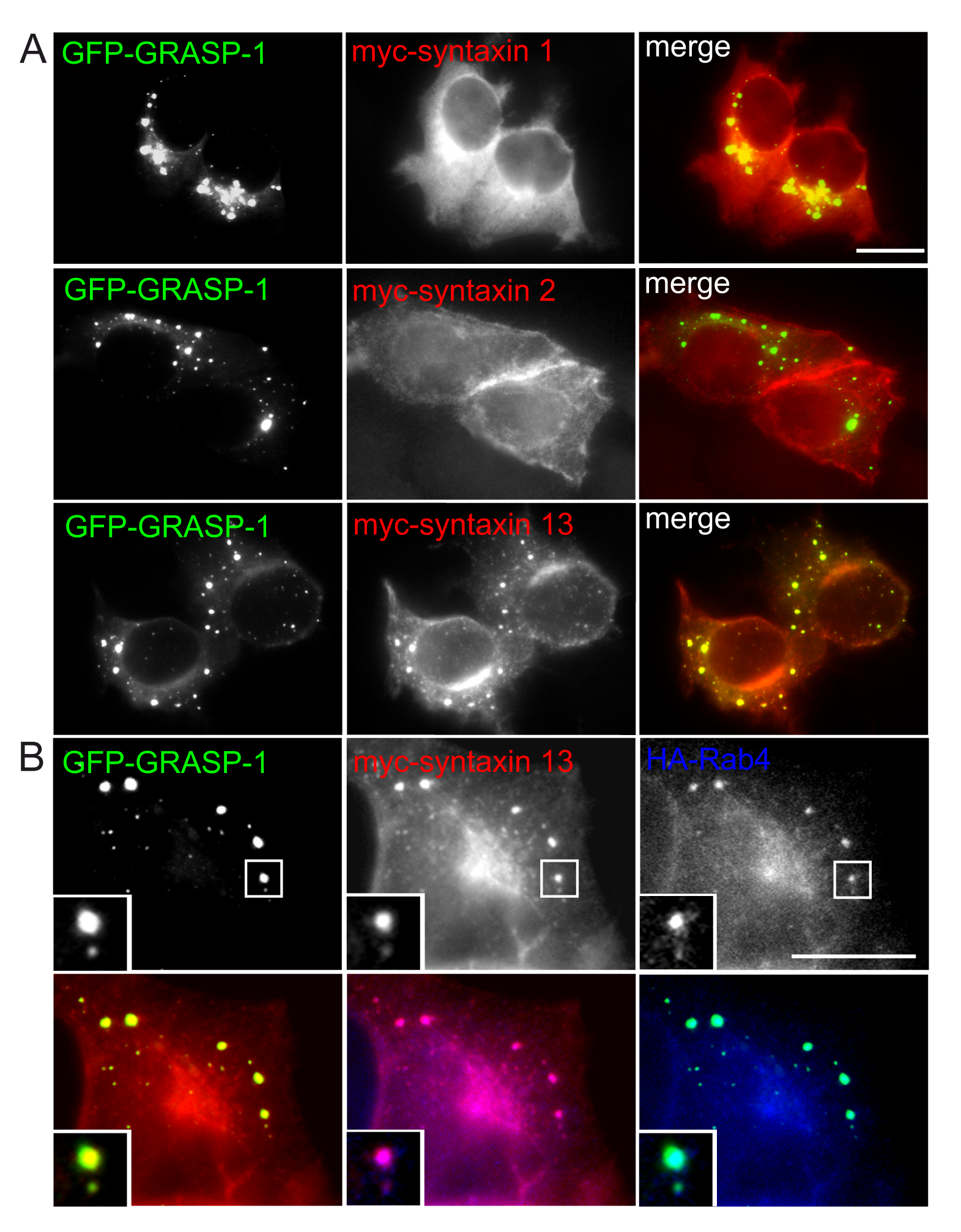

Supplement: Figure S9 — GRASP-1 coincides with Rab4 and syntaxin 13 in Hela cells. (A) Hela cells co-transfected with GFP-GRASP-1 and myc-syntaxin 1, myc-syntaxin 2, or myc-syntaxin 13. (B) Hela cells triple transfected with GFP-GRASP-1, myc-syntaxin 13, and HA-Rab4. Bar is 10 µm. (3.70 MB DOC) [file pbio.1000283.s009.tif]

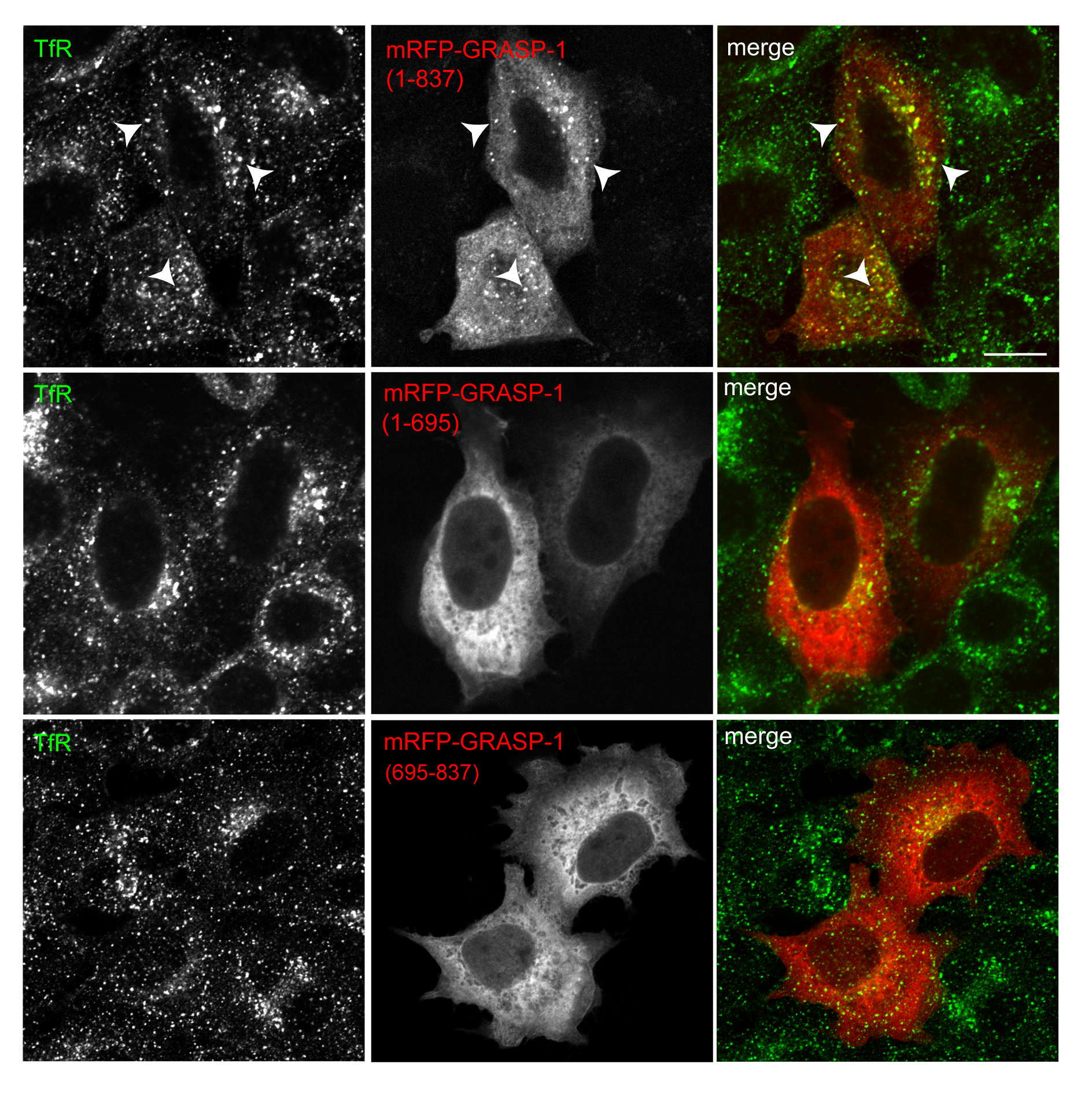

Supplement: Figure S10 — Both N and C terminus are necessary for GRASP-1 localization to endosomes. Hela cells transfected with full-length mRFP-GRASP-1 (1–837) or truncated mRFP-GRASP-1 constructs and labeled with anti-TfR antibodies (green). Bar is 10 µm. (5.64 MB TIF) [file pbio.1000283.s010.tif]
